# Colony Suppression and Possible Colony Elimination of the Subterranean Termites *Coptotermes formosanus* and *Reticulitermes speratus* by Discontinuous Soil Treatment Using a Diluent of Fipronil Suspension Concentrate

**DOI:** 10.3390/insects12040334

**Published:** 2021-04-08

**Authors:** Shuji Itakura, Johji Ohdake, Takashi Takino, Kiwamu Umezawa

**Affiliations:** 1 Department of Applied Biological Chemistry, Faculty of Agriculture, Kindai University, 3327-204 Naka-machi, Nara 631-8505, Japan; 2 Bayer Crop Science, 1-6-5, Marunouchi, Chiyoda-ku, Tokyo 100-8262, Japan; johji.ohdake@bayer.com; 3 Development Center, Bayer Crop Science, 9511-4, Yuki, Ibaraki 307-0001, Japan; takashi.takino@bayer.com

**Keywords:** colony elimination, colony suppression, discontinuous soil treatment, minimal use of insecticide, nonrepellent slow-acting termiticide

## Abstract

**Simple Summary:**

Termites play an important role in maintaining ecosystems, but they are also pests, exerting major economic impacts. Among the over 3000 known termite species, *Coptotermes* species and *Reticulitermes* species are the most common pest species. As one of the primary methods for controlling these subterranean termites, liquid termiticide is applied to the soil under and next to the building foundation to create a continuous chemical barrier. Nonrepellent slow-acting liquid termiticides, such as fipronil and imidacloprid, are used as the active ingredients in termite soil treatments. In the present study, to minimize the use of insecticides, a discontinuous soil treatment using fipronil was applied against subterranean termites, namely *Coptotermes formosanus* and *Reticulitermes speratus*, instead of the traditional continuous chemical barrier of termiticides. *C. formosanus* and *R. speratus* colonies were subjected to discontinuous soil treatments with fipronil and were strongly affected by the treatment at the colony level, resulting in colony suppression and possible colony elimination. Termite activity in the treated colony of *C. formosanus* was not found for more than two years, while that of *R. speratus* was not detected for three years.

**Abstract:**

We assessed the efficacy of a discontinuous soil treatment using a diluent of fipronil suspension concentrate in controlling colonies of *Coptotermes formosanus* and *Reticulitermes speratus*. In-ground monitoring stations were installed at Isogi Park and Kindai University, and individual termites inhabiting the stations were collected for four or six years to determine the numbers and locations of colonies present in test areas before and after the discontinuous soil treatment. Microsatellite genotyping indicated that two *C. formosanus* and two *R. speratus* colonies in the test area at Isogi Park and five *R. speratus* colonies in the test area at Kindai University were active and that their territories fluctuated every year. One of the two *C. formosanus* colonies at Isogi Park and one of the five *R. speratus* colonies at Kindai University were subjected to discontinuous soil treatments with fipronil and were strongly affected by the treatment at the colony level, resulting in the suppression and possible elimination of colonies. Termite activity of the fipronil-treated colony of *C. formosanus* was detected within one week after the discontinuous soil treatment and was not found for more than two years (28 months), while termite activity of the fipronil-treated colony of *R. speratus* was detected within four days and three weeks after the discontinuous soil treatment and was not detected thereafter for three years. Fipronil residue analysis showed that workers of *C. formosanus* moved at least 28 m and that workers of *R. speratus* moved 6 m from the treated soil locations for up to three weeks.

## 1. Introduction

Termites play an important role in maintaining ecosystems, with effects on the physical, chemical and biological properties of soils, soil microbiology, and plant growth [1,2,3]; however, they are also pests, exerting great economic impact worldwide. Subterranean termites are serious pests and are responsible for 80% of the annual global economic cost of termite damage and control, which reached US $40 billion in 2010 [4]. Subterranean termites account for 66 of 79 pest species and are considered serious pests among the over 3000 known termite species. Among the subterranean termites, 18 *Coptotermes* spp. are the most common structural pest species, followed by 10 *Reticulitermes* spp. and eight *Heterotermes* spp. [5]. Subsequent taxonomic and phylogeographic studies showed that *Coptotermes* species have been synonymized into 16 species [6].

The application of a liquid termiticide is one of the primary methods for controlling subterranean termites. The termiticide is applied to the soil under and next to the building foundation to create a continuous chemical barrier that prevents termites from entering a building. Nonrepellent slow-acting liquid termiticides have become a popular alternative to fast-acting repellent pyrethroids as the active ingredients in termite soil treatments [7]. The main active ingredients of nonrepellent slow-acting termiticides are fipronil, imidacloprid, chlorfenapyr, and chlorantraniliprole [8,9,10,11]. The effectiveness of these nonrepellent slow-acting termiticides against subterranean termites has been field-validated using the conventional soil treatment method in which a continuous exterior barrier of nonrepellent soil termiticides is established in soil on the structure’s exterior foundation walls. The following are reported as examples showing the effectiveness of these termiticides. All 11 of the fipronil-treated colonies of *Reticulitermes flavipes* (Kollar) disappeared within 90 days of treatment and were not found again [8]. Among the 11 imidacloprid-treated colonies of *R. flavipes* and the imidacloprid-treated colony of *Reticulitermes virginicus* (Banks), 75% disappeared within 90 days of treatment and were not detected again [12]. In the majority (83.3%) of the 12 chlorantraniprole-treated colonies of *R. flavipes*, termite activity was no longer detected within one or two months after the soil treatment and thereafter for two years [13].

Subterranean foraging galleries of a colony of *Coptotermes formosanus* Shiraki were excavated in a field, and multiple galleries ranging from 2 to 46 in (5 to 116 cm) depth were found [14]. In *C. formosanus*, 30 to 300 cm depth galleries were also excavated in a field [15]. Subterranean termites move through underground galleries instead of crawling on the surface of the ground; thus it is possible for termiticides to act on termites more efficiently by pouring termiticides into the ground instead of spraying them on the ground surface. For minimal use of insecticides, a discontinuous soil treatment using fipronil as a nonrepellent slow-acting termiticide was applied against subterranean termites in the present study instead of the traditional continuous chemical barrier of termiticides. In brief, a diluent of fipronil was poured into eight independent holes dug around a monitoring station infested by subterranean termites *C. formosanus* or *Reticulitermes speratus* (Kolbe). To assess accurately the effect of discontinuous soil treatment, termite activities were tracked by microsatellite genotyping over three years for *C. formosanus* and four years for *R. speratus* after the soil treatment.

## 2. Materials and Methods 

### 2.1. Monitoring and Collection of Termites

**Figure 1:**
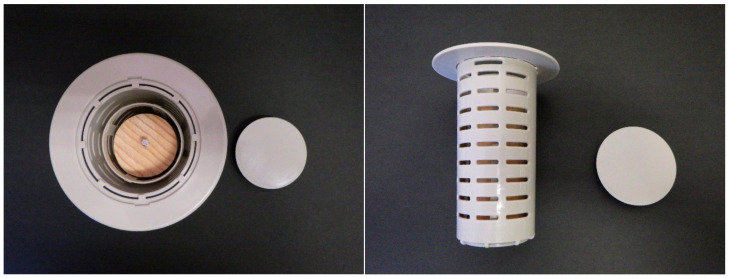
“Substec^TM^ station mini” with lid open and wood bait inside. **Left**: top view; **Right**: side view.

**Figure 2:**
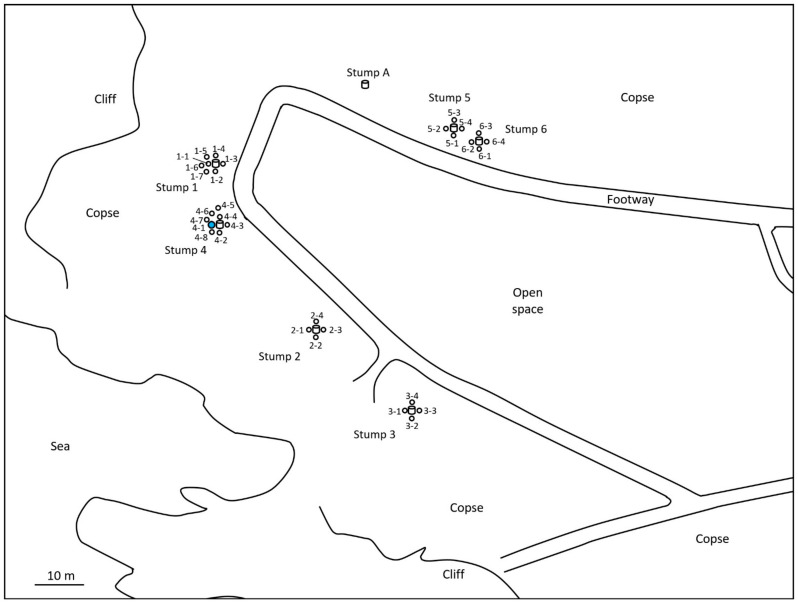
Location of monitoring stations (open circles) in Isogi Park. Twelve stations (1-1, 1-2, 1-3, 1-4, 2-1, 2-2, 2-3, 2-4, 3-1, 3-2, 3-3, and 3-4) were installed around stumps 1, 2, and 3 in June 2016. Four stations (4-1, 4-2, 4-3, and 4-4) were installed around stump 4 in July 2016. Three stations (1-5, 1-6, and 1-7) and four stations (4-5, 4-6, 4-7, and 4-8) were additionally installed around stumps 1 and 4, respectively, in December 2016. Eight stations (5-1, 5-2, 5-3, 5-4, 6-1, 6-2, 6-3, and 6-4) were installed around stumps 5 and 6 in March 2017. All stations were installed in-ground. Station 4-1, around which discontinuous soil treatment was carried out, is highlighted in blue.

**Figure 3:**
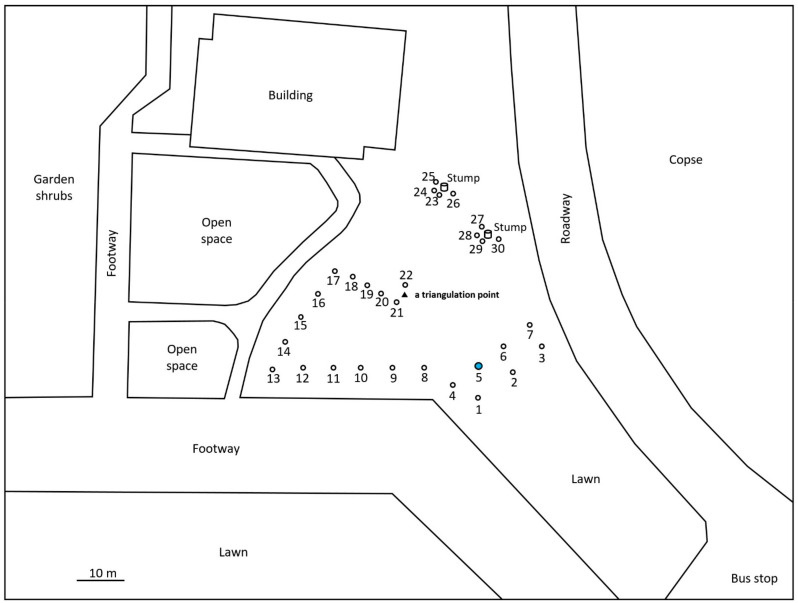
Location of monitoring stations (open circles) at Kindai University. All stations were installed in-ground in June 2013. Stations 1–7 were installed in flat ground. Stations 8–17 and 18–22 were set up at the foot of a hill and along the path, respectively. Stations 23–26 and 27–30 were installed around the stumps. Station 5, around which discontinuous soil treatment was carried out, is highlighted in blue.

For the monitoring and collection of *C. formosanus* and *R. speratus* termites, twelve “Substec^TM^ station mini” (SC Environmental Science, Osaka, Japan) stations containing wood were used for termite baiting (Figure 1) around three stumps (1, 2, and 3) at the test area in Isogi Park (33°39′35.7″ N, 135°20′34.4″ E, Wakayama Prefecture, Japan) in June 2016 (Figure 2). Four additional stations were installed around a fourth stump in July 2016. Three and four stations were additionally installed around stumps 1 and 4, respectively, in December 2016. Eight stations were installed around additional stumps, 5 and 6, in March 2017. For the monitoring and collection of *R. speratus* termites, thirty “Substec^TM^ station mini” stations containing wood were used for termite baiting at the test area on the Nara campus of Kindai University (34°40′18.5″ N, 135°44′03.4″ E, Nara Prefecture, Japan) in June 2013 (Figure 3). “Substec^TM^ wood mini” (SC Environmental Science, Osaka, Japan) wood inserts were used to monitor termite activity and were periodically inspected for four years, from 2016 to 2020, in Isogi Park and for six years, from 2013 to 2019, at Kindai University.

**Figure 4:**
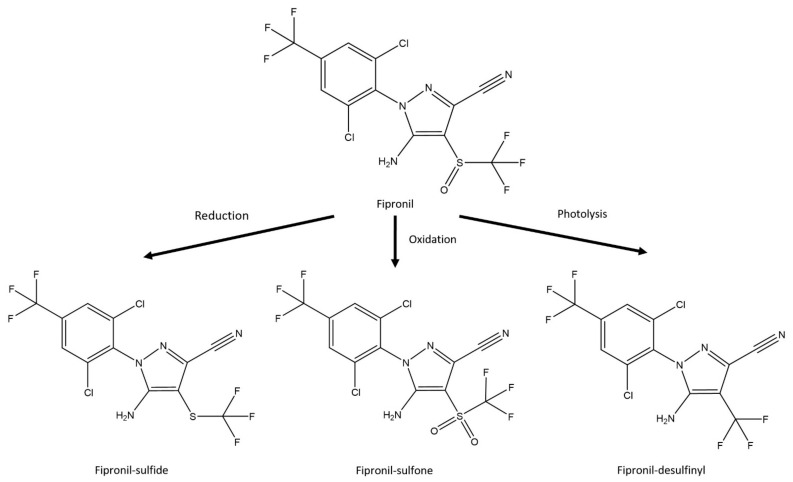
The degradation of fipronil to fipronil sulfide, fipronil sulfone, and fipronil desulfinyl occurs via reductive, oxidative, and photolytic mechanisms, respectively.

For microsatellite genotyping, between three and 19 workers and/or soldiers (*n* values, Table 1, Table 2 and Table 3) were collected from stations or stumps during inspection periods. For residue analysis of fipronil and its degradation products (Figure 4), between one and 14 *C. formosanus* foragers (*n* values, Table 4) were collected from Isogi Park stations one week after the soil treatment described below. Between five and 163 *R. speratus* foragers (*n* values, Table 5 and Table 6) were collected from the Kindai site four days and three weeks after discontinuous soil treatment with fipronil (see below). Specimens for both DNA genotyping and residue analysis were stored at −20 °C until extraction.

### 2.2. Discontinuous Soil Treatment

A discontinuous soil treatment was applied against subterranean termites in the present study instead of the traditional continuous chemical barrier of termiticides. A diluent of fipronil of a volume equivalent to one-third of the volume specified in the “banded spraying method” in the termite control standard specifications in Japan [16] was poured into eight independent holes dug at concentric circles with a radius of 20 or 30 cm from the monitoring stations infested by subterranean termites *C. formosanus* or *R. speratus*, respectively. 

In Isogi Park, worker and soldier caste termites of *C. formosanus* were found at stations 1-1, 2-2, 3-1, 4-1, 4-4, and 4-6 on 31 May 31 2017 (Figure 2). The next week, on 8 June, discontinuous soil treatment was carried out around station 4-1. Eight holes, with a diameter of 7.5 cm, a depth of 20 cm, and a volume of 883 cm^3^, were dug at concentric circles with a radius of 20 cm from station 4-1. After pouring 147 mL of Agenda^TM^ SC (9.1% fipronil suspension concentrate, Bayer CropScience, Tokyo, Japan) at the label rate of 0.03% into each hole, which is equivalent to one-third of the volume of 0.5 mL/cm^3^-soil specified in “banded spraying method” in the termite control standard specifications in Japan [16] assuming that the chemical treatment layer in the soil is 1 cm in depth, eight holes were backfilled with excavated soil. Because the distance between stations 4-1 and 4-4 was only 40 cm, if the radius was 30 cm, the distance between the hole poured with 0.03% Agenda^TM^ SC and station 4-4 would be only 10 cm; thus, the radius was set to 20 cm, which corresponded to the middle point between stations 4-1 and 4-4. In a survey at Kindai University on 26 October 2015, worker and soldier caste termites of *R. speratus* were found at stations 1, 2, 4, 5, 6, and 7 (Figure 3). The next week, on 5 November, discontinuous soil treatment was performed around station 5 in the same manner as described above, except that the radius of eight holes changed from 20 cm to 30 cm.

**Figure 5:**
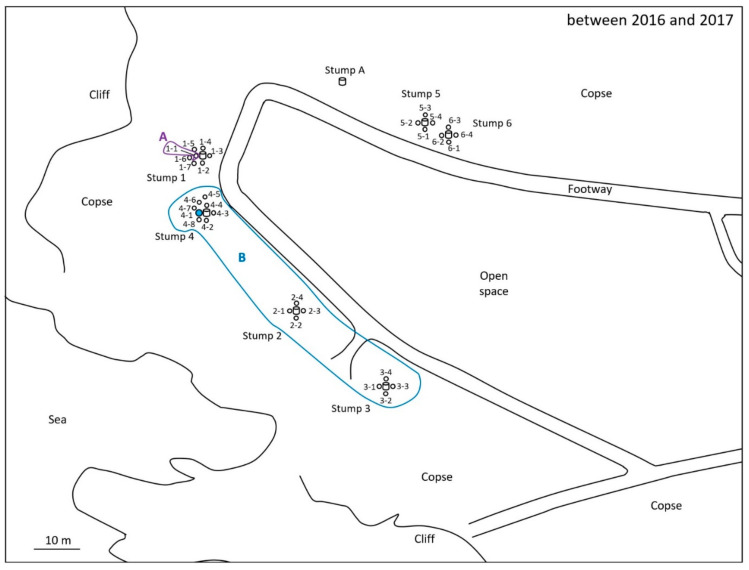

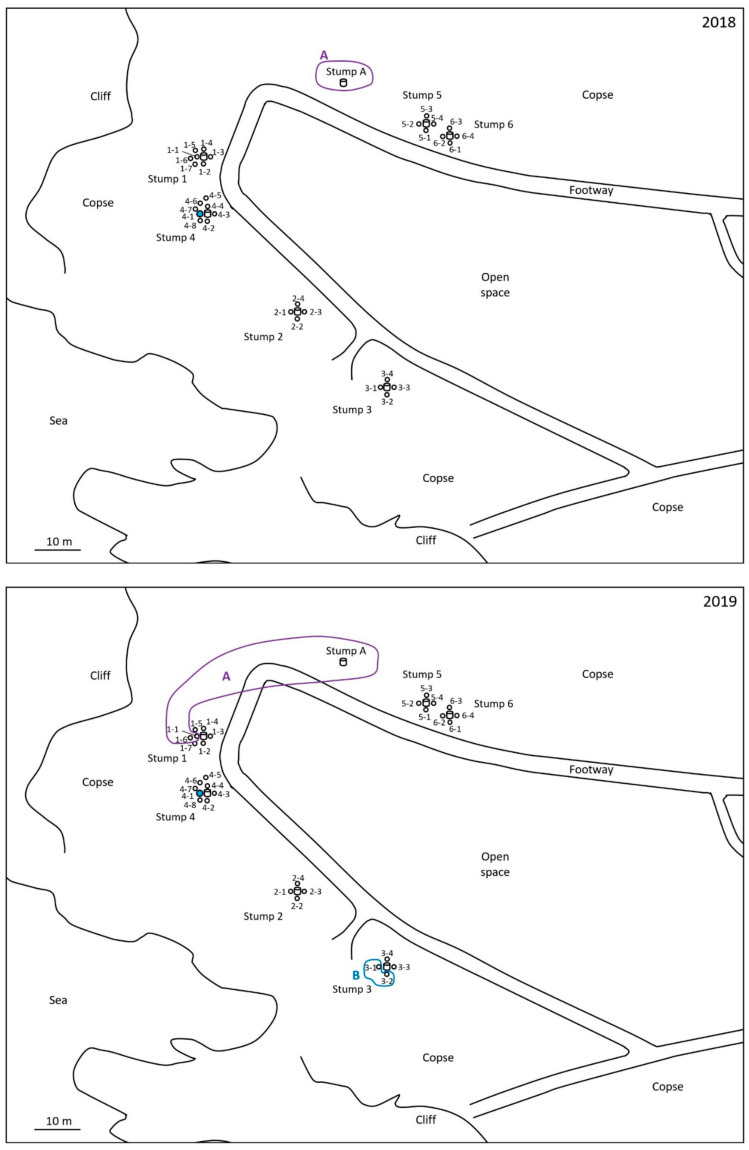

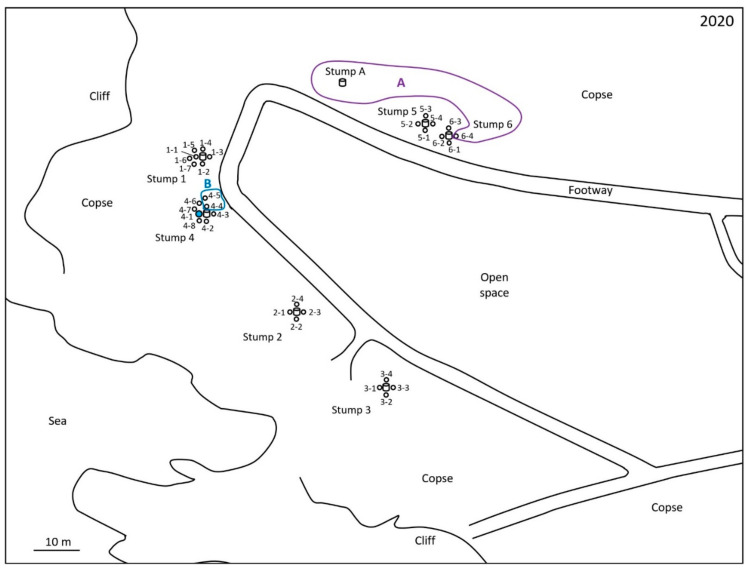
Location of monitoring stations occupied by *Coptotermes formosanus* in Isogi Park from 2016 to 2020. Stations with residing termites from the same population are encircled and labeled A and B. Station 4-1, around which discontinuous soil treatment was carried out, is highlighted in blue.

**Figure 6:**
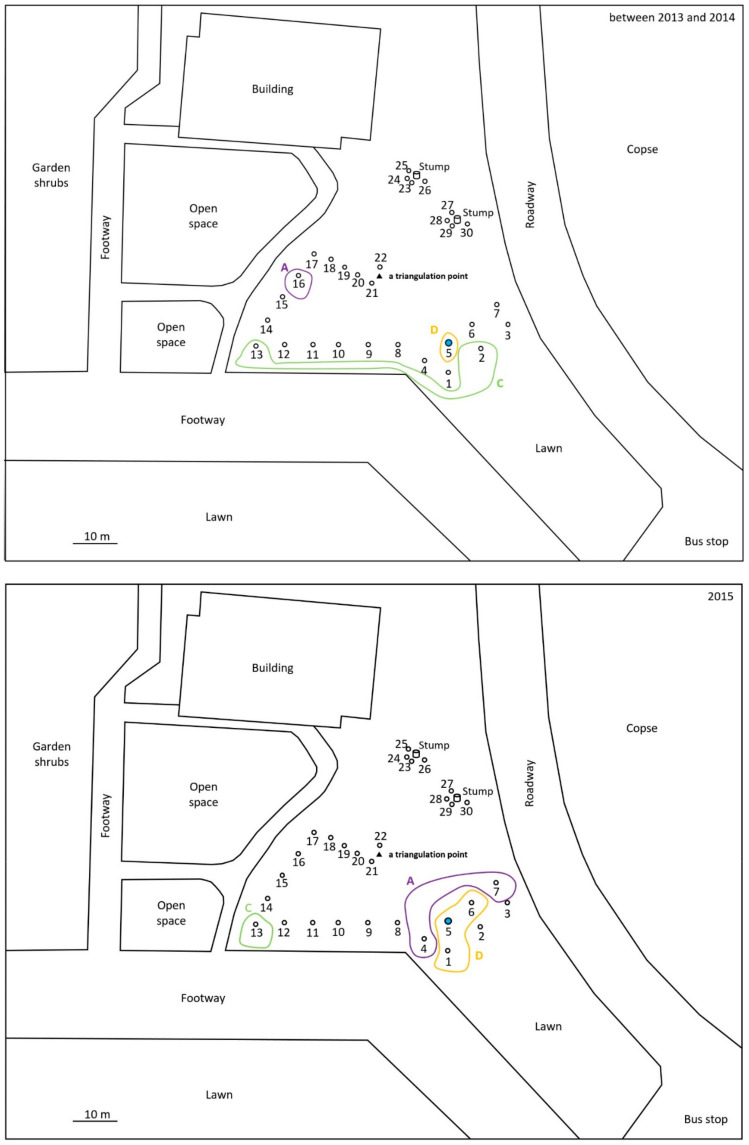

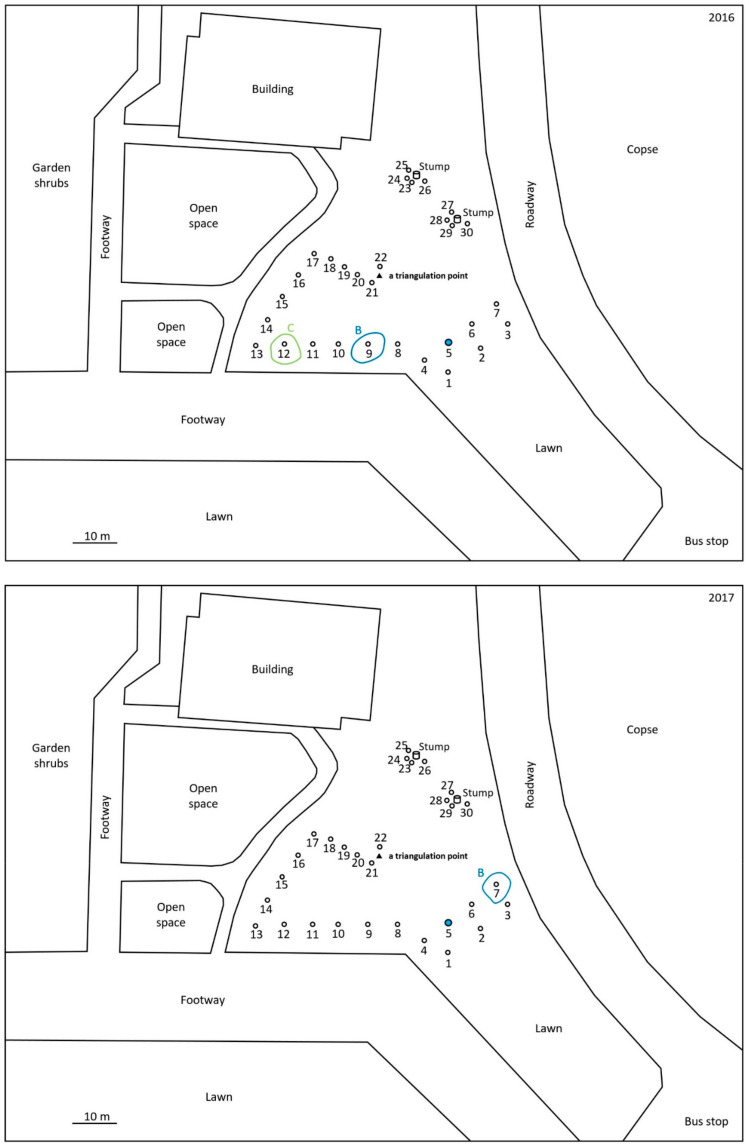

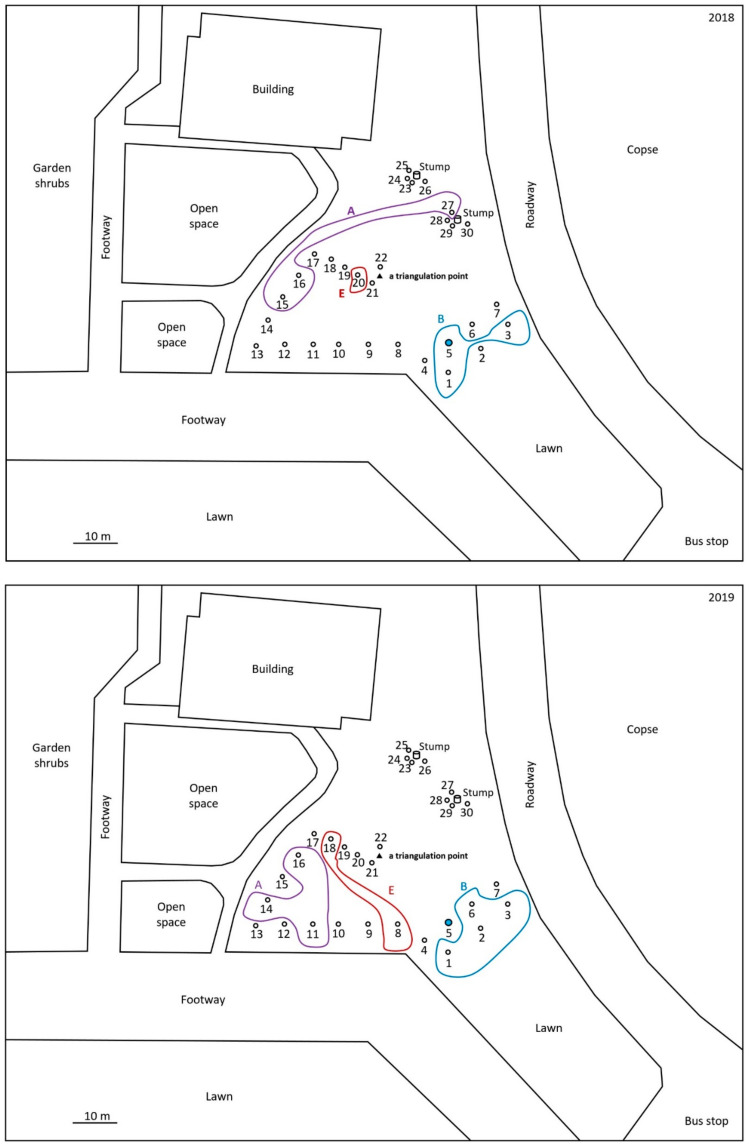
Location of monitoring stations occupied by *Reticulitermes speratus* at Kindai University from 2013 to 2019. Stations with residing termites from the same population are encircled and labeled A, B, C, D, and E. Station 5, around which discontinuous soil treatment was carried out, is highlighted in blue.

In order to verify the effectiveness of discontinuous soil treatment, individuals of the same colony need to inhabit multiple stations in the vicinity at the same time just before soil treatment; however, since such a situation rarely occurred during the test period of several years, the number of repetition was set to one for each of the two termite species. Untreated population A of *C. formosanus* at Isogi Park (Figure 5) and untreated populations A and C of *R. speratus* at Kindai University (Figure 6) were monitored as controls simultaneously with the monitoring of soil-treated colonies.

### 2.3. Determination of Fipronil and Its Derivatives on the Surface of Termites and in Termites

The weighed termites (values in parentheses shown in *n*, Table 4, Table 5 and Table 6) were rinsed with 10 mL of distilled water in a 30 mL Erlenmeyer flask, followed by filtration with glass wool. The rinsing and filtration were repeated, and the filtrates were combined to obtain a water fraction. Termites on glass wool were rinsed with 10 mL acetonitrile three times, and the filtrates were combined and evaporated under reduced pressure to obtain an acetonitrile fraction. Rinsed termites were homogenized in 5 mL acetonitrile solution, consisting of acetonitrile and water at a ratio of 80:20 (*v*/*v*), using a loosely fitting 5 mL Potter-Elvehjem homogenizer. Supernatant fluid of the homogenate was collected, while the precipitate was homogenized three more times in a similar manner in acetonitrile solution. The supernatant fluids (ca. 20 mL) were combined and centrifuged at 3000 rpm for 5 min. The supernatant separated by centrifugation was evaporated under reduced pressure to obtain termite body fractionation. Each fraction was purified with an SPE (solid phase extraction) column (Bond Elut C18, 500 mg, 3 mL, particle size 40 µm, Agilent Technologies, CA, USA). The water fraction was purified with an SPE column according to the following method. The SPE column was preconditioned with 2.5 mL acetonitrile and 2.5 mL water, sucked with a reduced presser. The water faction was passed through the column, sucked with a reduced presser, and then 5 mL acetonitrile solution, consisting of acetonitrile and water at a ratio of 40:60 (*v*/*v*), was passed through the column. Eluates were discarded. Finally, 15 mL acetonitrile solution, consisting of acetonitrile and water at a ratio of 60:40 (*v*/*v*), was passed through the column, and the eluate was collected. The acetonitrile fraction and termite body fraction were purified with an SPE column according to the following method. The SPE column was preconditioned with 2.5 mL acetonitrile and 2.5 mL acetonitrile solution, consisting of acetonitrile and water at a ratio of 40:60 (*v*/*v*). Each acetonitrile fraction and termite body fraction was dissolved in 2 mL of acetonitrile followed by the addition of 3 mL of water and mixing. Each solution was passed through the SPE column, sucked with a reduced presser, and then 5 mL acetonitrile solution, consisting of acetonitrile and water at a ratio of 40:60 (*v*/*v*), was passed through the column. Eluates were discarded. Finally, 15 mL acetonitrile solution, consisting of acetonitrile and water at a ratio of 60:40 (*v*/*v*), was passed through the column, and the eluate was collected. The eluates purified from the water fraction, acetonitrile fraction, and termite body fractionation were evaporated under reduced pressure and then dissolved in 0.5 mL acetonitrile solution, consisting of acetonitrile and water at a ratio of 50:50 (*v*/*v*), to be subjected to liquid chromatography-tandem mass spectrometry (LC-MS/MS) analysis. The LC-MS/MS analysis method was as follows. High-performance liquid chromatography (HPLC) analysis was performed online using a Shimadzu 20A series (Shimadzu, Kyoto, Japan). A Mightysil RP-18GP (150 mm, 2 mm ID, particle size 5 µm, Kanto Chemical, Tokyo, Japan) HPLC column was used with the mobile phase of acetonitrile and water at a ratio of 70:30 (*v*/*v*), pumped in isocratic mode at a flow rate of 0.2 mL/min. Electrospray ionization (ESI) tandem mass spectrometry was carried out in negative mode with a TSQ Vantage ™ triple stage quadrupole mass spectrometer (Thermo Fisher Scientific, MA, USA). The spray voltage was 3 kV, the vaporizer temperature was 450 °C, and the capillary temperature was 270 °C. Collision-induced dissociation MS/MS analysis, SRM (selected reaction monitoring), was conducted with argon as the collision gas, and the collision energy was adjusted to 24–33 eV to optimize the intensity of the daughter ion from the parent ion ([M–H]^−^) of each compound. The SRM parameters were shown in Table 7.

Detection limits for fipronil, fipronil-sulfide, fipronil-sulfone, and fipronil-desulfinyl were 0.5 ppb when a sufficient mass of termites (≥100 mg) was supplied for analysis. Detection limits (5 ppb maximum) increased inversely with termite mass (10 mg minimum) when supplied in insufficient quantities for analysis. The recovery rates of fipronil and its derivatives for each fraction were determined by performing similar analysis operations using termites with added 10 ppb standard reagents.

### 2.4. Microsatellite Genotyping for Termite Colony Affiliation

To assess accurately the effect of a nonrepellent slow-acting termiticide on termite colonies, it is necessary to distinguish the colonies present in the test area. Microsatellite genotyping was used to identify and track termite colonies and to evaluate the efficacy of bait [17,18] and soil termiticides [8,12,13]. Populations of *C. formosanus* at Isogi Park and *R. speratus* at Kindai University were first identified by microsatellite genotyping, and then each of the *C. formosanus* and *R. speratus* populations was treated with 0.03% fipronil by discontinuous soil treatment. The fates of populations were tracked over three years for *C. formosanus* and four years for *R. speratus* after the soil treatment.

Genomic DNA was extracted from the heads of workers using a DNeasy tissue kit (Qiagen, Hilden, Germany) according to the manufacturer’s recommended conditions. Termite head tissue was chosen to avoid contamination by residential gut microorganisms. Genomic DNA samples of *C. formosanus*, which were extracted from workers collected from 15 monitoring stations and four stumps in Isogi Park as well as workers collected from a laboratory population originally collected in Ohama Coast (33°43′14.0″ N 136°00′26.2″ E, Wakayama Prefecture, Japan) on 12 December 2012, were genotyped at four microsatellite loci: *Cf4-4*, *Cf4-9A*, *Cf8-4*, and *Cf10-5* [19]. Forward primers of *Cf4-4*, *Cf4-9A*, *Cf8-4*, and *Cf10-5* were labeled with fluorescent dyes FAM^TM^, VIC^TM^, NED^TM^, and PET^TM^, respectively (Applied Biosystems, Waltham, MA, USA). Genomic DNA samples of *R. speratus*, which were extracted from workers in Isogi Park and from workers collected from a laboratory population originally collected in Enjyu Coast (33°53′35.9″ N 135°07′57.4″ E, Wakayama Prefecture, Japan) on 12 November 2014, were genotyped at four microsatellite loci: *Rs02*, *Rs03*, *Rs05*, and *Rs07* [20]. Forward primers of *Rs02*, *Rs03*, *Rs05*, and *Rs07* were labeled with fluorescent dyes FAM^TM^, VIC^TM^, NED^TM^, and PET^TM^, respectively (Applied Biosystems, Waltham, MA, USA). Microsatellite loci were amplified using a Type-it Microsatellite PCR Kit (Qiagen, Hilden, Germany) and either a mixture of the forward and reverse primers of *Rs02*, *Rs03*, *Rs05*, and *Rs07* for *R. speratus* or a mixture of the forward and reverse primers of *Cf4-4*, *Cf4-9A*, *Cf8-4*, and *Cf10-5* for *C. formosanus* according to the manufacturer’s protocol. The PCR conditions were 30 cycles of 30 s at 95 °C, 90 s at 60 °C, and 30 s at 72 °C [21]. PCR products were resolved on an ABI Prism 3100 Genetic Analyzer (Applied Biosystems, Waltham, MA, USA). Genotypes were scored by comparing the fluorescently labeled PCR products to size standards, GeneScan^TM^ 500 LIZ Size Standard (Applied Biosystems, Waltham, MA, USA), by using Peak Scanner Software v 1.0 (Applied Biosystems, Waltham, MA, USA). The number of alleles, allele frequency, and genotypes of the cohorts determined by means of a genetic differentiation exact G test were compared using GenePop on the web (https://genepop.curtin.edu.au/, accessed on 5 January 2021). The expected and observed heterozygosities, Wright’s *F*-statistics *F*_ST_, *F*_IT_, and *F*_IS_ [22], and the genetic distance of cohorts were calculated using the genetic data analysis (GDA) program (http://lewis.eeb.uconn.edu/lewishome/software.html, accessed on 3 September 2010) [23]. Dendrograms obtained by the GDA program were visualized using TreeView v 1.6.6 (http://taxonomy.zoology.gla.ac.uk/rod/treeview.html, accessed on 3 September 2010).

## 3. Results

**Figure 7:**
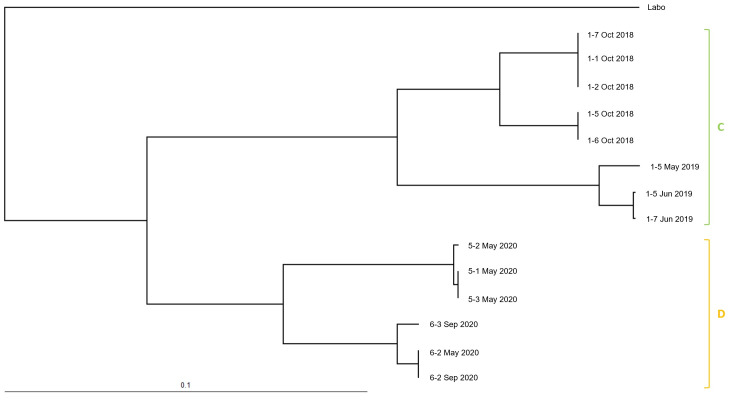
Dendrogram obtained using UPGMA (unweighted pair group method with arithmetic mean) among cohorts of *Reticulitermes speratus* collected in Isogi Park. Cohorts from the same population are labeled C and D.

**Figure 8:**
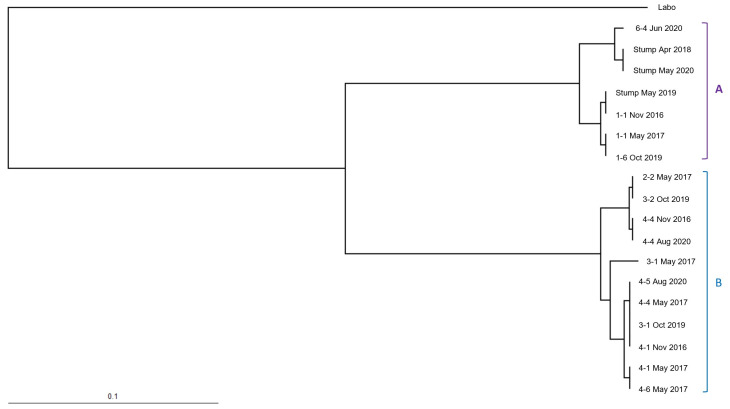
Dendrogram obtained using UPGMA between cohorts of *Coptotermes formosanus* collected in Isogi Park. Cohorts from the same population are labeled A and B.

**Figure 9:**
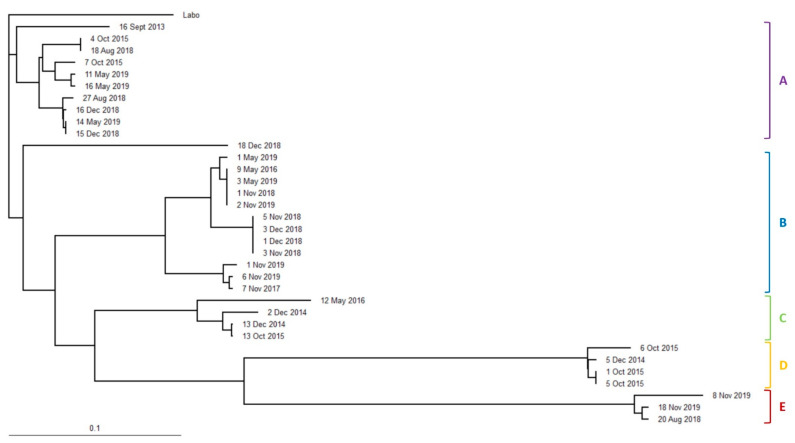
Dendrogram obtained using UPGMA among cohorts of *Reticulitermes speratus* collected at Kindai University. Cohorts from the same population are labeled A, B, C, D and E.

In total, 14 cohorts from two populations of *R. speratus* (Figure 7) and 18 cohorts from two populations of *C. formosanus* in Isogi Park (Figure 8) (of which 9 cohorts had been found at the time of discontinuous soil treatment (Table 1)) and 34 cohorts of *R. speratus* in Kindai University (Figure 9) (of which 10 cohorts had been found at the time of discontinuous soil treatment (Table 3)) were detected. As shown in Table 1, Table 2 and Table 3, the number of alleles of *C. formosanus* at four loci, *Cf4-4*, *Cf4-9A*, *Cf8-4*, and *Cf10-5*, and that of *R. speratus* at four loci, *Rs02*, *Rs03*, *Rs05*, and *Rs07*, did not exceed four in all cohorts, suggesting that these cohorts may be simple family colonies (groups of cohabiting individuals produced by a monogamous pair of reproductives) or extended family colonies (groups of cohabiting individuals produced by multiple inbred neotenic reproductives descended from the original founding pair) but not mixed family colonies (groups of cohabiting individuals produced by multiple unrelated reproductives) [24]. The *F*_IS_, the coefficient of inbreeding for individuals within their subpopulations, is a statistic that is sensitive to the numbers of reproductives present. The *F*_IS_ shows a strong negative value (approximately −0.3) in a simple family colony, while in extended family colonies, the *F*_IS_ shows negative values (approximately −0.15) and rises towards zero as the number of reproductives per generation increases. In mixed family colonies, the *F*_IS_ shows positive values under simulated breeding systems [25]. The average *F*_IS_ of *C. formosanus* was −0.319 ± 0.009 in Isogi Park (Table 1), which was consistent with that in a simple family colony, while the average *F*_IS_ of *R. speratus* was −0.061 ± 0.014 in Isogi Park (Table 2), which was consistent with that in extended family colonies with many neotenics (10 to 100), and −0.145 ± 0.012 in Kindai University (Table 3), which was consistent with that in extended family colonies with low numbers of neotenics (less than 10) under simulated breeding systems [24]. The *F*_IT_, the coefficient of inbreeding for individuals relative to the total population, shows positive values in extended family colonies and mixed family colonies, except for a simple family colony in which the *F*_IT_ is zero under simulated breeding systems [25]. The average *F*_IT_ of *C. formosanus* was −0.020 ± 0.006 in Isogi Park (Table 1), which was close to zero and was almost consistent with that in a simple family colony. The average *F*_IT_ of *R. speratus* was 0.196 ± 0.017 in Isogi Park (Table 2) and 0.212 ± 0.008 in Kindai University (Table 3), which were positive values and consistent with those in extended family colonies. The *F*_ST_, the coefficient of inbreeding of subpopulations relative to the total population, shows positive values in a simple family colony, extended family colonies, and mixed family colonies under simulated breeding systems [25]. The average *F*_ST_ of *C. formosanus* was 0.227 ± 0.002 in Isogi Park (Table 1), which was consistent with that in a simple family colony, while that of *R. speratus* was 0.231 ± 0.012 in Isogi Park (Table 2) and 0.314 ± 0.005 in Kindai University (Table 3), which was consistent with that in extended family colonies. Although the average observed heterozygosity (*H*o) was greater than the average expected heterozygosity (*H*e) in *C. formosanus* and *R. speratus*, no significant difference was observed (*p* > 0.05) between *H*o and *H*e (Table 1, Table 2 and Table 3).

**Figure 10:**
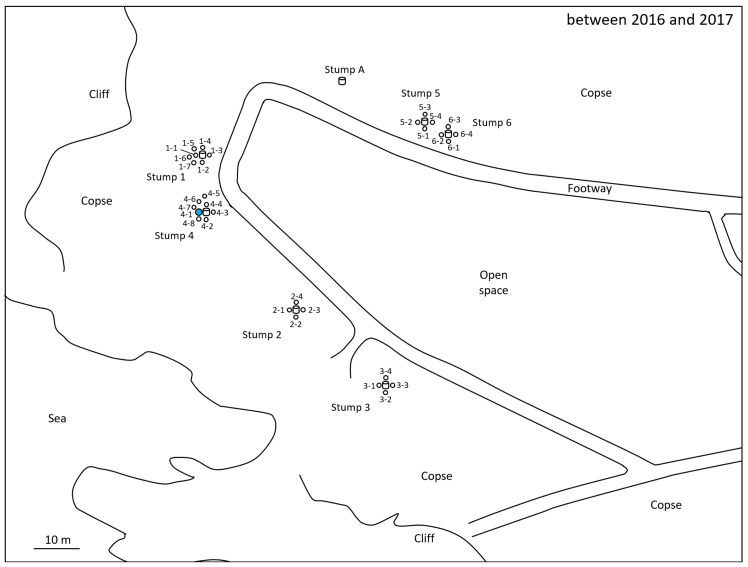

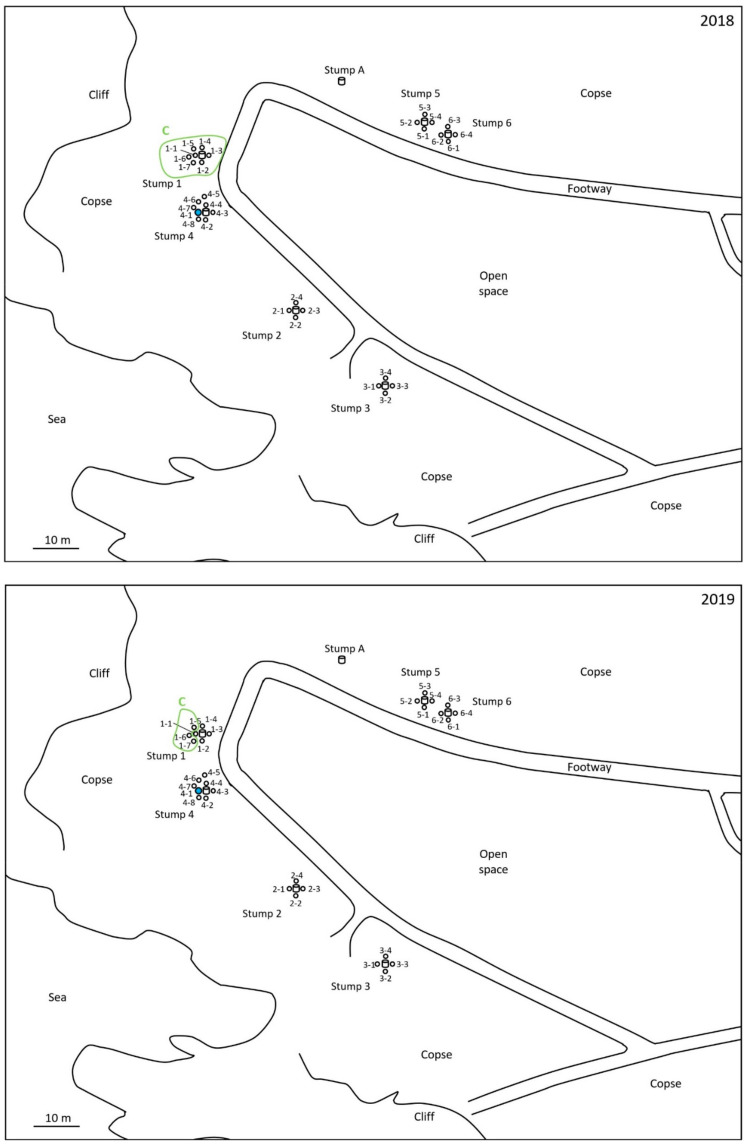

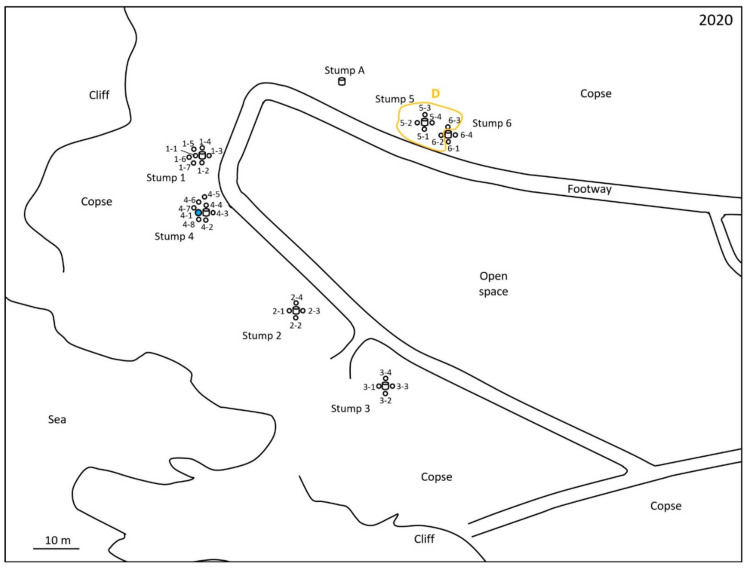
Location of monitoring stations occupied by *Reticulitermes speratus* in Isogi Park from 2016 to 2020. Stations with residing termites from the same population are encircled and labeled C and D. Station 4-1, around which discontinuous soil treatment was carried out, is highlighted in blue.

The UPGMA (unweighted pair group method with arithmetic mean) dendrograms are presented in Figure 7, Figure 8 and Figure 9. Cohorts of *C. formosanus* in Isogi Park were clustered into two colonies (Figure 8, A and B), while populations of *R. speratus* in Isogi Park were also clustered into two populations (Figure 7, C and D). Most cohorts of *R. speratus* at Kindai University, except for the cohort “18 December 2018”, were clustered into five colonies (Figure 9, A to E). Based on clustering in the UPGMA dendrogram, the transitions of population activities of *C formosanus* and *R. speratus* in Isogi Park and that of *R. speratus* in Kindai University are shown in Figure 5, Figure 6, and Figure 10.

For *C. formosanus* in Isogi Park, discontinuous soil treatment was performed around station 4-1, a cohort of which belonged to population B, on 31 May 2017 (Figure 5). No *C. formosanus* was found in the monitoring stations in the survey one month after the discontinuous soil treatment on 6 July 2017. Subsequent surveys at 3, 5, 8, 11, 12, 13.5, 15, 16, and 21 months later, in 2017 and 2018, found no *C. formosanus* termites belonging to population B at the monitoring stations; however, workers and soldiers of *C. formosanus* belonging to population A were collected from stump A on 25 April, 13 June, and 1 September 2018. In the survey on 21 October 2019, twenty-eight months after the discontinuous soil treatment, worker and soldier termites of *C. formosanus* were collected from monitoring station 1-6, which belonged to population A, and from monitoring stations 3-1 and 3-2, which belonged to population B (Figure 5). No *C. formosanus* termites were found in the monitoring stations in the survey on 1 January 2020, thirty-one months after the discontinuous soil treatment. In the survey on 8 June 2020, thirty-six months after the discontinuous soil treatment, workers and soldiers of *C. formosanus* were collected from the monitoring station 6-4, which belonged to population A. Then, in the survey on 6 August 2020, thirty-eight months after the discontinuous soil treatment, workers and soldiers of *C. formosanus* were collected from the monitoring stations 4-4 and 4-5, which belonged to population B. No *C. formosanus* termites were found in the monitoring stations, but they were found in stump A, which belonged to population A, in the final survey on 8 September 2020, thirty-nine months after the discontinuous soil treatment. In brief, the untreated population A of *C. formosanus* at Isogi Park continued to persist throughout the study in Isogi Park from 2016 to 2020 (Figure 5).

Around stump 1 in Isogi Park, monitoring station 1-1 was inhabited by *C. formosanus* on 1 November 2016 and 31 May 2017 (Figure 5). *R. speratus* settled at monitoring stations 1-1 and 1-6 on 1 September 2018, at monitoring stations 1-1, 1-2, 1-5, 1-6, and 1-7 on 26 October 2018, and at monitoring stations 1-5, 1-6, and 1-7 on 6 March 2019 (Figure 10). After that, *C. formosanus* returned and was damaging monitoring station 1-6 on 21 October 2019. Similar reinvasions of vacating foraging territory caused by using the chitin synthesis inhibitor with neighboring colonies were often observed in *C. formosanus* and *R. flavipes* [18,26]. Temporal and spatial segregation by termites was also observed around stump 6. *C. formosanus* was found at monitoring station 6-4 on 8 June 2020. Two months later, *R. speratus* was damaging monitoring station 6-2 on 6 August 2020, and one month later, the monitoring stations 6-2 and 6-3 were being damaged on 8 September 2020 (Figure 5 and Figure 10).

For *R. speratus* at Kindai University, discontinuous soil treatment was performed around station 5, a cohort of which belonged to population D, on 5 November 2015 (Figure 6). No termites inhabited monitoring stations 1, 2, 3, 4, 5, 6, and 7 in the survey on 12 May 2016, six months after the discontinuous soil treatment (Figure 6). Monitoring station 5, which had been treated with fipronil, was free from *R. speratus* termites from May 2016 to August 2018, approximately 27 months. Monitoring station 5 was reinfested by *R. speratus* on 5 November 2018, three years after the discontinuous soil treatment. Microsatellite genotyping data revealed that individuals inhabiting monitoring station 5 in October 2015 belonged to population D, but that those in station 5 in November 2018 belonged to population B. Population B first appeared at monitoring station 9 in May 2016 and continued to exist in 2017, 2018 and 2019. Population B was observed at monitoring station 7 on the other side of the test area across monitoring station 5 in 2017, and the range of population B expanded to a width of approximately 16.5 m, from station 1 to station 3, in 2018. At the time of the discontinuous soil treatment, monitoring stations 4 and 7, which belonged to population A, would not be directly connected to stations 1, 5, and 6, which belonged to population D, by underground termite galleries. Station 4 was 6 m away from soil-treated station 5; similarly, monitoring station 7 was 13 m away from station 5. Population A, which disappeared in 2016 and 2017 and reappeared in 2018 and 2019 in the test area, would not have been eliminated by the discontinuous soil treatment with fipronil around station 5 because population A was not directly connected to station 5 and was more than 6 m away from station 5. These results are consistent with the results that 60% of untreated colonies, which were more than 6 m away from the foundation wall treated with fipronil, survived for 3 years after the treatment [8]. Termites belonging to population D were collected from monitoring station 5 on 9 December 2014 and from monitoring stations 1, 5, and 6 on 26 October 2015, before the discontinuous soil treatment. *R. speratus* termites belonging to population D were never collected since they were last collected on 26 November 2015, which corresponds to three weeks after the soil treatment. Although monitoring station 5 was reinfested by *R. speratus* three years after the discontinuous soil treatment, the reinfesting termites were shown to belong to another population, B, by microsatellite analysis. As mentioned above, population A, which was adjacent to the soil-treated station 5 but not directly connected, would not have been eliminated by the discontinuous soil treatment with fipronil because population A reappeared in 2018 and 2019 in the test area (Figure 6). Similarly, untreated population C continued to persist until May 2016 (Table 3, Figure 6). In brief, untreated population C continued to persist for approximately half a year after the discontinuous soil treatment, and untreated population A was not eliminated from the test area at Kindai University by the discontinuous soil treatment over three years.

As shown in Table 4, fipronil and fipronil-sulfone (oxidative metabolite) were detected in the termite body fraction of workers of *C. formosanus* collected from monitoring station 2-2 one week after the discontinuous soil treatment on 15 June 2017 (Figure 5, population B). In a soldier caste termite collected from monitoring station 4-4, fipronil and fipronil-sulfone were detected in the water and acetonitrile fractions obtained by rinsing termites but not in the termite body fraction (Table 4). Fipronil-desulfinyl (photodegradation product) was detected in the acetonitrile fraction of a soldier collected from monitoring station 4-4. No fipronil, fipronil-sulfide (reductive metabolite), fipronil-sulfone, or fipronil-desulfinyl was detected in the water, acetonitrile, or termite body fractions of the two soldiers collected from monitoring station 2-2. As shown in Table 5, fipronil and fipronil-sulfone were detected in the termite body fraction of workers of *R. speratus* collected from monitoring stations 1, 5, and 6 (Figure 6, population D) four days after the discontinuous soil treatment, while fipronil-sulfone was detected in the water and acetonitrile fractions obtained by rinsing termites collected from monitoring stations 1 and 5 and in the acetonitrile fraction of monitoring station 6. However, fipronil and fipronil-sulfone were not detected in termites collected from monitoring station 4, where termites belonged to population A; this was different from population D (Figure 6). No termites were collected at monitoring stations 2, 3, and 7 four days after the discontinuous soil treatment. At the collection three weeks after the discontinuous soil treatment on 26 November 2015, no fipronil was detected in the termite body fraction, as well as the water and acetonitrile fractions, of individuals collected from monitoring stations 1, 5, and 6, while fipronil-sulfone was detected in the termite body fraction of individuals collected from monitoring stations 1, 5, and 6 (Table 6). Fipronil-sulfone was also detected in the water and acetonitrile fractions of termites collected from monitoring stations 1 and 6. No termites were collected at monitoring stations 2, 3, 4, and 7 three weeks after the discontinuous soil treatment. Fipronil-sulfide and fipronil-desulfinyl were not detected in the water, acetonitrile, or termite body fractions of workers of *R. speratus* collected four days and three weeks after the discontinuous soil treatment. The recovery rates of fipronil and its derivatives were 92–97% in the water fraction, 83–101% in the acetonitrile fraction, and 97–103% in the termite body fraction.

## 4. Discussion

The average *F*_IS_ of population A and that of population B of *C. formosanus* were −0.322 ± 0.012 and −0.317 ± 0.006, respectively, while the average *F*_IS_ of population A and population B combined was −0.319 ± 0.009 in Isogi Park (Table 1). Similarly, the average *F*_IT_ and average *F*_ST_ of each population were not significantly different from those of all colonies in *C. formosanus*. The average *F*_IS_ of each of population A and population B, as well as that of the total of both colonies, was consistent with that in a simple family colony under the simulated breeding systems [24,25]. Differences in the allele sizes of the two colonies, A and B, analyzed by microsatellite genotyping, showed that two simple family colonies (A and B) would be found by different pairs of kings and queens derived from winged alates in Isogi Park (Supplementary Table S4, 212 bp in locus Cf4-4, 285 bp in locus Cf4-9A, 221 bp in locus Cf8-4, and 265 bp in locus Cf10-5). In *C. formosanus* in Hawaii and Louisiana, both simple families (headed by a pair of reproductives) and extended families (headed by varying numbers of inbreeding reproductives) were found by microsatellite genotyping [27]. Three colonies out of 30 colonies of *C. formosanus* in Kyushu and Fukue were reported as extended families having begun as simple families but being headed by multiple neotenic (secondary) reproductives descending from the original king and queen [17]. Later division and isolation of a colony with a pair of male and female reproductives from headed colonies by multiple neotenic reproductives could give a simple family colony.

The average *F*_IS_ of population C and that of population D of *R. speratus* were −0.053 ± 0.012 and −0.072 ± 0.009, respectively, while the average *F*_IS_ of the total of population C and population D was −0.061 ± 0.014 in Isogi Park (Table 2). The average *F*_IS_ of each of population C and population D, as well as that of the total of both colonies, was consistent with that in extended family colonies with many neotenics (10 to 100) [24] and a colony mated with ten female and ten male neotenics with three generations [25] under the simulated breeding systems. The average *F*_IS_ of population A, B, C, D, and E of *R. speratus* were −0.146 ± 0.008, −0.145 ± 0.001, −0.145 ± 0.035, −0.141 ± 0.004, and −0.149 ± 0.011, respectively, while the average *F*_IS_ of the total of colonies A, B, C, D, and E was −0.145 ± 0.012 at Kindai University (Table 3). Similarly, the average *F*_IT_ and average *F*_ST_ of each population were not significantly different from those of the total of all colonies in *R. speratus*. Each average *F*_IS_ was consistent with that in extended family colonies with low numbers of neotenics (less than 10) [24] and colonies mated with one or two female neotenics and one male neotenic with three generations [25] under the simulated breeding systems. The *F*_IS_ of mixed colonies consisting of genetically complex groups from unrelated nests would be positive under simulated breeding systems [25]. Negative *F*_IS_ values of *R. speratus* in Isogi Park and Kindai University showed that these colonies could not occur as a fusion of different colonies, though colony merging occurs naturally in *R. speratus* [28] as well as other *Reticulitermes* sp. [29,30]. Although it has been reported that *R. speratus* females reproduce parthenogenetically and find colonies cooperatively with partner females or even alone [31], cohorts collected in Isogi Park and Kindai University do not reproduce parthenogenetically because the occurrence of neotenic parthenogenesis in the field has never been known [32].

Subterranean foraging galleries of a colony of *C. formosanus* were excavated in a field, and galleries more than 200 feet (61 m) in length were found [14]. In Isogi Park, the distance between monitoring station 4-1, around which soil was treated discontinuously with fipronil, and monitoring station 2-2 was approximately 28 m (Figure 2). Detection of fipronil and fipronil-sulfone from workers of *C. formosanus* collected from monitoring station 2-2 a week after the discontinuous soil treatment (Table 4) showed that the workers taking fipronil into the body near monitoring station 4-1 moved 28 m to monitoring station 2-2 in a week. At Kindai University, monitoring stations 1 and 5, as well as stations 5 and 6, were installed at an interval of 6 m. Fipronil and fipronil-sulfone were detected in the termite body fraction of workers of *R. speratus* collected from monitoring stations 1, 5, and 6 four days after the discontinuous soil treatment (Table 5). Fipronil-sulfone was also detected in the termite body fraction of workers collected from monitoring stations 1, 5, and 6 at collection three weeks after the discontinuous soil treatment (Table 6). These results showed that workers of *C. formosanus* moved at least 28 m and that workers of *R. speratus* moved at least 6 m after contacting fipronil-treated soil around the stations, although termites exposed to fipronil had been reported to never move more than 5 m [33].

As mentioned above, fipronil and fipronil-sulfone were detected from workers of *C. formosanus* collected a week after the discontinuous soil treatment and from workers of *R. speratus* collected four days after the discontinuous soil treatment; in addition, fipronil-sulfone was also detected from workers collected three weeks after the discontinuous soil treatment. These results showed that workers of *C. formosanus* crawled in galleries or tunnels for at least one week and that workers of *R. speratus* crawled in galleries or tunnels for at least three weeks. These time durations were important for fipronil to be distributed among nestmates.

Fipronil-desulfinyl was only detected in the acetonitrile fraction of a soldier of *C. formosanus* collected from monitoring station 4-4 (Table 4) but was not detected in workers. Soldiers have a role of vigilance. After the soil treatment, soldiers with fipronil attached to cuticle surfaces could be crawling around the surface of the ground and/or wood for vigilance, and it is possible that they will be exposed to light during alert behavior. This vigilant action seems to be the reason why fipronil-desulfinyl was detected only in the acetonitrile fraction of the soldier as fipronil-desulfinyl is a photodegradation product of fipronil.

In Isogi Park, workers and soldiers of *C. formosanus* were collected from monitoring station 2-2 and a soldier was collected from monitoring station 4-4 a week after the discontinuous soil treatment on 15 June 2017 (Figure 5, population B). After that, *C. formosanus* termites belonging to population B, which had been treated with fipronil, had not been found for approximately twenty-eight months; on 21 October 2019, worker and soldier termites of *C. formosanus* were collected from monitoring stations 3-1 and 3-2, which belonged to population B. Though no *C. formosanus* termites belonging to population B were observed in the subsequent 10-month survey, *C. formosanus* termites belonging to population B were observed and collected again on 6 August 2020, thirty-eight months after the discontinuous soil treatment. To summarize briefly, *C. formosanus* termites, which were determined to belong to population B, reinvaded to the area where population B originally occupied, approximately 28 months later. Although the discontinuous soil treatment was able to weaken the population for 28 months, it seems that population B of *C. formosanus* could not be eradicated by the discontinuous soil treatment. However, microsatellite genotyping analysis of *C. formosanus* in Maui showed that the genetic diversity of populations in the introduced colonies (Maui and Kauai, Hawaii) was lower than that in the native colonies (Guangdong and Hunan, China), and that subpopulations headed by nest mate pairs as alates or neotenics from the same colony inhabited the same area [34]. Experiments in planar arenas showed that a neighboring colony of *C. formosanus* reinfested the territory in which another population of *C. formosanus* was previously eliminated by baiting [35]. In the present study in Isogi Park, colony B, which existed at the time of the discontinuous soil treatment in 2017, and colony B, to which termites collected from stations 3-1 and 3-2 in 2019 and from stations 4-4 and 4-5 in 2020 belonged, may have been different subcolonies headed from the same colony, as reported in the introduced colonies in Maui. Because the cohorts identified as population B in 2017 and 2019 came from different subcolonies, which had been branched from the same colony, it is estimated that the population B that existed in 2017 could have been eradicated by discontinuous soil treatment and that another population B, which was determined to belong to the same population B as the fipronil-treated population B in 2017 by microsatellite genotyping, could have reinfested the test area in 2019.

At Kindai University, workers and soldiers of *R. speratus* belonging to population D were collected from monitoring stations 1, 4, 5, and 6 four days after the discontinuous soil treatment on 9 November 2015 and from monitoring stations 1, 5, and 6 three weeks after the discontinuous soil treatment on 26 November 2015. Although no termites were collected from station 5 in 2016 and 2017, station 5 was reinfested by *R. speratus* belonging to population B on 5 November 2018 (just three years after the discontinuous soil treatment). *R. speratus* belonging to population D was not detected again. The above results indicate that the discontinuous soil treatment successfully eliminated population D of *R. speratus* from the test area at Kindai University.

In the conventional soil treatment method, a continuous exterior barrier of nonrepellent soil termiticides, such as fipronil, chlorantraniliprole, and imidacloprid, is established in soil on the structure’s exterior foundation walls. A full treatment or exterior/localized interior treatment with fipronil at the label rate of 0.06% against the eastern subterranean termite *R. flavipes* caused strong colony suppression, probably resulting in colony elimination [8]. Exterior/localized interior treatment with other nonrepellent soil termiticides, 0.05% chlorantraniliprole [13] and 0.05% imidacloprid [12], caused suppression and apparent elimination of *R. flavipes* and *R. virginicus* colonies. In the present study, 0.03% fipronil was poured into eight discontinuous holes arranged concentrically with a radius of 20 cm from monitoring station 4-1 in Isogi Park for *C. formosanus* and 30 cm from monitoring station 5 in Kindai University for *R. speratus* instead of building a continuous barrier. The discontinuous soil treatment using 0.03% fipronil successfully suppressed the activity of *C. formosanus* termites for 28 months and *R. speratus* termites for approximately 42 months, and it seems that the colony was eradicated in *R. speratus*. However, the number of replication for each of the two termite species was only one in the present study, so it is necessary to repeat field tests in future studies to verify the effectiveness of the discontinuous soil treatment against the subterranean termites.

## Figures and Tables

**Table 1:** Statistics of microsatellite loci and *F*-statistics for *Coptotermes formosanus* cohorts collected in Isogi Park from November 2016 to May 2020.

Cohort ^1^		Population	*n*	*N*_A_				*F*_IS_	*F*_IT_	*F*_ST_	*H*e	*H*o
Station No	Collection Date			*Cf4-4*	*Cf4-9A*	*Cf8-4*	*Cf10-5*					
**1-1**	**Nov 2016**	A	10	1	1	2	2	−0.340	−0.035	0.226	0.250	0.250
**1-1**	**May 2017**	A	11	2	1	2	2	−0.312	−0.017	0.225	0.292	0.409
1-6	Oct 2019	A	10	2	1	2	2	−0.330	−0.025	0.230	0.272	0.275
**2-2**	**May 2017**	B	6	2	2	2	2	−0.314	−0.016	0.227	0.337	0.458
**3-1**	**May 2017**	B	8	2	2	2	2	−0.318	−0.013	0.232	0.388	0.500
3-1	Oct 2019	B	10	2	2	1	2	−0.315	−0.014	0.229	0.324	0.450
3-2	Oct 2019	B	10	2	2	1	2	−0.317	−0.017	0.228	0.286	0.375
**4-1**	**Nov 2016**	B	12	2	2	1	2	−0.314	−0.012	0.229	0.322	0.438
**4-1**	**May 2017**	B	10	2	2	1	2	−0.306	−0.012	0.224	0.339	0.500
**4-4**	**Nov 2016**	B	16	2	2	1	2	−0.329	−0.026	0.228	0.201	0.234
**4-4**	**May 2017**	B	3	2	2	1	2	−0.316	−0.020	0.225	0.367	0.500
4-4	Aug 2020	B	6	2	2	1	2	−0.322	−0.021	0.228	0.280	0.333
4-5	Aug 2020	B	4	2	2	1	2	−0.322	−0.022	0.227	0.277	0.313
**4-6**	**May 2017**	B	6	2	2	1	2	−0.312	−0.018	0.224	0.314	0.458
6-4	Jun 2020	A	8	2	1	2	2	−0.322	−0.025	0.226	0.371	0.438
Stump A	Apr 2018	A	17	2	1	2	2	−0.328	−0.024	0.230	0.313	0.368
	Mar 2019	A	8	1	1	2	2	−0.311	−0.018	0.225	0.246	0.313
	May 2020	A	8	2	1	2	2	−0.309	−0.017	0.223	0.338	0.500
Laboratory			10	2	2	2	2	−0.309	−0.103	0.158	0.397	0.550
Mean ± SD (without laboratory)			9.1 ± 3.6	1.9 ± 0.3	1.6 ± 0.5	1.5 ± 0.5	2.0 ± 0	−0.319 ± 0.009	−0.020 ± 0.006	0.227 ± 0.002	0.306 ± 0.048	0.395 ± 0.090

^1^ Cohort: notated as a combination of station number plus collection date; bold: cohorts found by the time of discontinuous soil treatment; *n*: sample size; *N*_A_: number of alleles; *F*_IS_: coefficient of inbreeding for individuals within their subpopulations; *F*_IT_: coefficient of inbreeding for individuals relative to the total population; *F*_ST_: coefficient of inbreeding of subpopulations relative to the total population; *H*e: expected heterozygosity; *H*o: observed heterozygosity.

**Table 2:** Statistics of microsatellite loci and *F*-statistics for *Reticulitermes speratus* cohorts collected in Isogi Park from October 2018 to September 2020.

Cohort ^1^		Population	*n*	*N*_A_				*F*_IS_	*F*_IT_	*F*_ST_	*H*e	*H*o
Station No	Collection Date			*Rs02*	*Rs03*	*Rs05*	*Rs07*					
1-1	Oct 2018	C	8	2	1	2	1	−0.054	0.204	0.233	0.248	0.406
1-2	Oct 2018	C	8	2	1	2	2	−0.058	0.202	0.243	0.292	0.375
1-5	Oct 2018	C	7	2	1	2	2	−0.061	0.198	0.228	0.332	0.321
1-5	Mar 2019	C	8	2	2	2	1	−0.025	0.221	0.231	0.367	0.625
1-5	Jun 2019	C	11	2	2	2	1	−0.053	0.225	0.246	0.348	0.545
1-6	Oct 2018	C	8	2	1	2	2	−0.060	0.200	0.213	0.348	0.344
1-7	Oct 2018	C	8	2	1	2	2	−0.060	0.200	0.242	0.273	0.344
1-7	Jun 2019	C	7	2	2	2	2	−0.049	0.215	0.246	0.442	0.607
5-1	May 2020	D	8	3	2	1	1	−0.078	0.185	0.232	0.208	0.219
5-2	May 2020	D	9	3	1	1	1	−0.062	0.184	0.223	0.165	0.250
5-3	May 2020	D	8	3	1	1	1	−0.067	0.183	0.225	0.169	0.219
6-2	May 2020	D	8	3	1	1	1	−0.086	0.166	0.224	0.158	0.063
6-2	Sep 2020	D	8	3	1	1	1	−0.073	0.176	0.224	0.177	0.188
6-3	Sep 2020	D	8	3	1	1	1	−0.067	0.179	0.223	0.127	0.156
Laboratory			12	3	4	3	2	−0.109	0.110	0.203	0.569	0.646
Mean ± SD (without laboratory)			8.1 ± 0.9	2.4 ± 0.5	1.3 ± 0.5	1.6 ± 0.5	1.4 ± 0.5	−0.061 ± 0.014	0.196 ± 0.017	0.231 ± 0.012	0.261 ± 0.097	0.333 ± 0.169

^1^ Cohort: notated as a combination of station number plus collection date; *n*: sample size; *N*_A_: number of alleles; *F*_IS_: coefficient of inbreeding for individuals within their subpopulations; *F*_IT_: coefficient of inbreeding for individuals relative to the total population; *F*_ST_: coefficient of inbreeding of subpopulations relative to the total population; *H*e: expected heterozygosity; *H*o: observed heterozygosity.

**Table 3:** Statistics of microsatellite loci and *F*-statistics for *Reticulitermes speratus* cohorts collected at Kindai University from September 2013 to November 2019.

Cohort ^1^		Population	*n*	*N*_A_				*F*_IS_	*F*_IT_	*F*_ST_	*H*e	*H*o
Station No	Collection Date			*Rs02*	*Rs03*	*Rs05*	*Rs07*					
**1**	**Oct 2015**	D	9	2	3	1	1	−0.138	0.213	0.312	0.301	0.444
1	Nov 2018	B	8	1	2	1	1	−0.146	0.211	0.313	0.100	0.125
1	Dec 2018	B	8	1	2	1	1	−0.146	0.211	0.314	0.131	0.156
1	May 2019	B	12	1	2	1	1	−0.146	0.207	0.310	0.040	0.042
1	Nov 2019	B	11	3	2	1	1	−0.142	0.215	0.316	0.205	0.250
**2**	**Dec 2014**	C	12	3	3	1	2	−0.112	0.229	0.312	0.438	0.458
2	Nov 2019	B	12	2	2	1	1	−0.145	0.211	0.313	0.119	0.146
3	Nov 2018	B	8	1	2	1	1	−0.144	0.213	0.313	0.125	0.188
3	Dec 2018	B	8	1	2	1	1	−0.146	0.211	0.314	0.131	0.156
3	May 2019	B	12	2	2	1	1	−0.145	0.211	0.313	0.126	0.146
**4**	**Oct 2015**	A	14	3	3	2	1	−0.154	0.211	0.320	0.307	0.321
**5**	**Dec 2014**	D	12	2	3	1	1	−0.146	0.207	0.312	0.257	0.271
**5**	**Oct 2015**	D	17	2	3	1	1	−0.138	0.210	0.309	0.291	0.382
5	Nov 2018	B	8	1	2	1	1	−0.144	0.213	0.313	0.125	0.188
6	Oct 2015	D	19	2	3	1	1	−0.143	0.197	0.299	0.209	0.250
6	Nov 2019	B	10	2	2	1	1	−0.142	0.213	0.314	0.158	0.200
**7**	**Oct 2015**	A	4	3	3	3	2	−0.162	0.205	0.317	0.580	0.438
7	Nov 2017	B	12	2	2	1	1	−0.144	0.210	0.312	0.093	0.104
8	Nov 2019	E	8	2	2	2	1	−0.142	0.213	0.312	0.273	0.344
9	May 2016	B	12	2	2	1	1	−0.146	0.210	0.312	0.093	0.104
11	May 2019	A	11	3	2	2	1	−0.137	0.222	0.318	0.315	0.386
12	May 2016	C	12	2	2	2	2	−0.145	0.198	0.302	0.414	0.396
**13**	**Dec 2014**	C	6	2	3	1	2	−0.130	0.219	0.313	0.345	0.375
**13**	**Oct 2015**	C	18	4	2	1	2	−0.194	0.197	0.324	0.251	0.250
14	May 2019	A	12	3	2	2	1	−0.144	0.221	0.322	0.369	0.396
15	Dec 2018	A	8	3	2	2	1	−0.141	0.218	0.317	0.390	0.438
**16**	**Sept 2013**	A	14	2	3	1	1	−0.150	0.209	0.315	0.299	0.286
16	Dec 2018	A	8	3	2	2	1	−0.147	0.215	0.318	0.290	0.313
16	May 2019	A	11	3	2	2	1	−0.150	0.216	0.321	0.328	0.295
18	Aug 2018	A	11	3	2	2	1	−0.144	0.219	0.320	0.341	0.364
18	Dec 2018	-	8	3	2	1	2	−0.130	0.224	0.316	0.433	0.563
18	Nov 2019	E	11	3	1	1	1	−0.143	0.206	0.307	0.124	0.136
20	Aug 2018	E	10	3	1	1	2	−0.162	0.202	0.315	0.253	0.175
27	Aug 2018	A	11	3	2	2	1	−0.134	0.225	0.318	0.331	0.523
Laboratory			12	3	4	3	2	−0.151	0.193	0.299	0.569	0.646
Mean ± SD (without laboratory)			10.8 ± 3.2	2.3 ± 0.8	2.2 ± 0.5	1.4 ± 0.5	1.2 ± 0.4	−0.145 ± 0.012	0.212 ± 0.008	0.314 ± 0.005	0.252 ± 0.126	0.283 ± 0.133

^1^ Cohort: notated as a combination of station number plus collection date; bold: cohorts found by the time of discontinuous soil treatment; *n*: sample size; *N*_A_: number of alleles; *F*_IS_: coefficient of inbreeding for individuals within their subpopulations; *F*_IT_: coefficient of inbreeding for individuals relative to the total population; *F*_ST_: coefficient of inbreeding of subpopulations relative to the total population; *H*e: expected heterozygosity; *H*o: observed heterozygosity.

**Table 4:** Fipronil and its sulfide, sulfone, and desulfinyl derivatives detected in worker and soldier caste termites of *Coptotermes formosanus* collected in Isogi Park one week after the soil treatment.

Station No./Caste	*n*	Sample	Fipronil	Fipronil-Sulfide (Reductive Metabolite)	Fipronil-Sulfone (Oxidative Metabolite)	Fipronil-Desulfinyl (Photodegradation Product)	Total
			(pg/termite) ^2^				
2-2/Worker	14 (52.3 mg)	Water fraction	0.0	0.0	0.0	0.0	0.0
(0.5 ppb, 1.8 pg/termite) ^1^		Acetonitrile fraction	0.0	0.0	0.0	0.0	0.0
		Termite body fraction	1.9	0.0	4.5	0.0	6.4
		Total	1.9	0.0	4.5	0.0	6.4
2-2/Soldier	2 (8.4 mg)	Water fraction	0	0	0	0	0
(3 ppb, 12 pg/termite) ^1^		Acetonitrile fraction	0	0	0	0	0
		Termite body fraction	0	0	0	0	0
		Total	0	0	0	0	0
4-4/Soldier	1 (3.4 mg)	Water fraction	21	0	23	0	44
(6 ppb, 21 pg/termite) ^1^		Acetonitrile fraction	32	0	38	162	231
		Termite body fraction	0	0	0	0	0
		Total	53	0	60	162	275

^1^ Detection limit for each sample. ^2^ Values below the detection limits are shown as 0 even if trace amounts were quantified. *n*: sample size, with weight of termites in parentheses.

**Table 5:** Fipronil and its sulfide, sulfone, and desulfinyl derivatives detected from worker caste termites of *Reticulitermes speratus* collected at Kindai University four days after the soil treatment.

Station No.	*n*	Sample	Fipronil	Fipronil-Sulfide (Reductive Metabolite)	Fipronil-Sulfone (Oxidative Metabolite)	Fipronil-Desulfinyl (Photodegradation Product)	Total
			(pg/termite) ^2^				
1	163 (268.3 mg)	Water fraction	0.0	0.0	1.6	0.0	1.6
(0.5 ppb, 0.8 pg/termite) ^1^		Acetonitrile fraction	0.0	0.0	1.5	0.0	1.5
		Termite body fraction	1.8	0.0	12.6	0.0	14.4
		Total	1.8	0.0	15.8	0.0	17.6
4	13 (26.8 mg)	Water fraction	0	0	0	0	0
(2 ppb, 4 pg/termite) ^1^		Acetonitrile fraction	0	0	0	0	0
		Termite body fraction	0	0	0	0	0
		Total	0	0	0	0	0
5	59 (124.1 mg)	Water fraction	0.0	0.0	3.3	0.0	3.3
(0.5 ppb, 1.1 pg/termite) ^1^		Acetonitrile fraction	0.0	0.0	3.1	0.0	3.1
		Termite body fraction	24.8	0.0	90.0	0.0	114.8
		Total	24.8	0.0	96.4	0.0	121.2
6	48 (84.2 mg)	Water fraction	0.0	0.0	0.0	0.0	0.0
(0.6 ppb, 1.1 pg/termite) ^1^		Acetonitrile fraction	0.0	0.0	1.3	0.0	1.3
		Termite body fraction	1.4	0.0	9.7	0.0	11.1
		Total	1.4	0.0	11.0	0.0	12.4

^1^ Detection limit for each sample. ^2^ Values below the detection limits are shown as 0 even if trace amounts were quantified. *n*: sample size, with weight of termites in parentheses.

**Table 6:** Fipronil and its sulfide, sulfone, and desulfinyl derivatives detected from worker caste termites of *Reticulitermes speratus* collected at Kindai University three weeks after the soil treatment.

Station No.	*n*	Sample	Fipronil	Fipronil-Sulfide (Reductive Metabolite)	Fipronil-Sulfone (Oxidative Metabolite)	Fipronil-Desulfinyl (Photodegradation Product)	Total
			(pg/termite) ^2^				
1	135 (294 mg)	Water fraction	0.0	0.0	2.2	0.0	2.2
(0.5 ppb, 1.1 pg/termite) ^1^		Acetonitrile fraction	0.0	0.0	3.2	0.0	3.2
		Termite body fraction	0.0	0.0	18.0	0.0	18.0
		Total	0.0	0.0	23.4	0.0	23.4
5	5 (9.3 mg)	Water fraction	0	0	0	0	0
(5 ppb, 9.3 pg/termite) ^1^		Acetonitrile fraction	0	0	0	0	0
		Termite body fraction	0	0	30	0	30
		Total	0	0	30	0	30
6	71 (117.6 mg)	Water fraction	0.0	0.0	1.6	0.0	1.6
(0.5 ppb, 0.8 pg/termite) ^1^		Acetonitrile fraction	0.0	0.0	2.1	0.0	2.1
		Termite body fraction	0.0	0.0	4.2	0.0	4.2
		Total	0.0	0.0	7.9	0.0	7.9

^1^ Detection limit for each sample. ^2^ Values below the detection limits are shown as 0 even if trace amounts were quantified. *n*: sample size, with weight of termites in parentheses.

**Table 7:** Selected reaction monitoring (SRM) parameters at MS/MS analysis.

Compound	Parent Ion (*m*/*z*)	Daughter Ion (*m*/*z*)	Collision Energy (eV)	S-Lens
Fipronil	435	250	32	96
Fipronil-Sulfide	419	262	33	87
Fipronil-Sulfone	451	282	31	104
Fipronil-Desulfinyl	387	351	24	77

## Data Availability

Detailed data are available upon request.

## Supplementary Materials

The following are available online at https://www.mdpi.com/article/10.3390/insects12040334/s1, Table S1: Sizes of alleles detected in cohorts of *Coptotermes formosanus* in Isogi Park, Table S2: Sizes of alleles detected in cohorts of *Reticulitermes speratus* in Isogi Park, Table S3: Sizes of alleles detected in cohorts of *Reticulitermes speratus* at Kindai University, Table S4: Allele frequency in each locus of *Coptotermes formosanus* in Isogi Park, Table S5: Allele frequency in each locus of *Reticulitermes speratus* in Isogi Park, Table S6: Allele frequency in each locus of *Reticulitermes speratus* at Kindai University.
