# Supplementary material for: Colony Suppression and Possible Colony Elimination of the Subterranean Termites Coptotermes formosanus and Reticulitermes speratus by Discontinuous Soil Treatment Using a Diluent of Fipronil Suspension Concentrate
Source: Insects. 2021 Apr 8;12(4):334. doi: 10.3390/insects12040334 (PMC8068406; doi:10.3390/insects12040334)
Supplement: Supplementary file 1 [file insects-12-00334-s001.zip › TableS1.docx]

**Table S1:** Sizes of alleles detected in cohorts of *Coptotermes formosanus* in Isogi Park.

| Cohort | Alleles (bp) | | | |
| --- | --- | --- | --- | --- |
|  | Cf4-4 | Cf4-9A | Cf8-4 | Cf10-5 |
| 1-1_Nov_2016 | | | | |
| 1 | 215/215 | 282/282 | 221/227 | 271/283 |
| 2 | 215/215 | 282/282 | 227/227 | 271/283 |
| 3 | 215/215 | 282/282 | 227/227 | 271/283 |
| 4 | 215/215 | 282/282 | 227/227 | 283/283 |
| 5 | 215/215 | 282/282 | 221/221 | 271/283 |
| 6 | 215/215 | 282/282 | 221/221 | 271/271 |
| 7 | 215/215 | 282/282 | 227/227 | 271/283 |
| 8 | 215/215 | 282/282 | 221/227 | 283/283 |
| 9 | 215/215 | 282/282 | 221/227 | 271/283 |
| 10 | 215/215 | 282/282 | 227/227 | 271/283 |
|  |  |  |  |  |
| 1-1_May_2017 | | | | |
| 1 | 215/218 | 282/282 | 221/227 | 271/283 |
| 2 | 215/215 | 282/282 | 221/227 | 271/283 |
| 3 | 215/215 | 282/282 | 221/227 | 271/283 |
| 4 | 215/218 | 282/282 | 221/227 | 271/283 |
| 5 | 215/215 | 282/282 | 227/227 | 271/283 |
| 6 | 215/215 | 282/282 | 227/227 | 271/271 |
| 7 | 215/215 | 282/282 | 227/227 | 271/283 |
| 8 | 215/215 | 282/282 | 227/227 | 271/271 |
| 9 | 215/218 | 282/282 | 221/227 | 271/283 |
| 10 | 215/215 | 282/282 | 227/227 | 271/283 |
| 11 | 215/215 | 282/282 | 221/227 | 271/283 |
|  |  |  |  |  |
| 1-6_Oct_2019 | | | | |
| 1 | 215/218 | 282/282 | 227/227 | 283/283 |
| 2 | 215/215 | 282/282 | 221/227 | 271/271 |
| 3 | 215/215 | 282/282 | 221/227 | 283/283 |
| 4 | 215/215 | 282/282 | 227/227 | 271/283 |
| 5 | 215/215 | 282/282 | 227/221 | 271/283 |
| 6 | 215/215 | 282/282 | 227/221 | 271/271 |
| 7 | 215/215 | 282/282 | 221/227 | 283/283 |
| 8 | 215/218 | 282/282 | 227/227 | 283/283 |
| 9 | 215/215 | 282/282 | 227/227 | 271/283 |
| 10 | 215/215 | 282/282 | 227/227 | 271/283 |
|  |  |  |  |  |
| 2-2_May_2017 | | | | |
| 1 | 215/215 | 282/285 | 227/227 | 283/283 |
| 2 | 215/215 | 282/282 | 227/227 | 265/283 |
| 3 | 215/215 | 282/285 | 227/227 | 265/283 |
| 4 | 215/215 | 282/285 | 227/227 | 283/283 |
| 5 | 212/215 | 282/285 | 224/227 | 265/283 |
| 6 | 215/215 | 282/285 | 227/227 | 265/283 |
|  |  |  |  |  |
| 3-1_May_2017 | | | | |
| 1 | 215/215 | 282/285 | 227/227 | 283/283 |
| 2 | 215/215 | 282/282 | 227/227 | 283/283 |
| 3 | 212/215 | 282/285 | 224/227 | 265/283 |
| 4 | 212/215 | 282/282 | 224/227 | 283/283 |
| 5 | 212/215 | 282/285 | 224/227 | 283/283 |
| 6 | 212/215 | 282/285 | 224/227 | 283/283 |
| 7 | 212/215 | 282/285 | 227/227 | 265/283 |
| 8 | 215/215 | 282/282 | 227/227 | 283/283 |
|  |  |  |  |  |
| 3-1_Oct_2019 | | | | |
| 1 | 212/215 | 282/285 | 227/227 | 265/283 |
| 2 | 215/215 | 282/285 | 227/227 | 283/283 |
| 3 | 215/215 | 282/282 | 227/227 | 265/283 |
| 4 | 212/215 | 282/285 | 227/227 | 265/283 |
| 5 | 212/215 | 282/282 | 227/227 | 265/283 |
| 6 | 215/215 | 282/285 | 227/227 | 265/283 |
| 7 | 212/215 | 282/285 | 227/227 | 283/283 |
| 8 | 215/215 | 282/282 | 227/227 | 265/283 |
| 9 | 212/215 | 282/282 | 227/227 | 265/283 |
| 10 | 215/215 | 282/282 | 227/227 | 265/283 |
|  |  |  |  |  |
| 3-2_Oct_2019 | | | | |
| 1 | 215/215 | 282/285 | 227/227 | 283/283 |
| 2 | 215/215 | 282/285 | 227/227 | 265/283 |
| 3 | 215/215 | 282/285 | 227/227 | 283/283 |
| 4 | 215/215 | 282/285 | 227/227 | 265/283 |
| 5 | 215/215 | 282/282 | 227/227 | 283/283 |
| 6 | 212/215 | 282/285 | 227/227 | 265/283 |
| 7 | 215/215 | 282/282 | 227/227 | 265/283 |
| 8 | 212/215 | 282/282 | 227/227 | 265/283 |
| 9 | 215/215 | 282/285 | 227/227 | 283/283 |
| 10 | 212/215 | 282/285 | 227/227 | 283/283 |
|  |  |  |  |  |
| 4-1_Nov_2016 | | | | |
| 1 | 215/215 | 282/285 | 227/227 | 283/283 |
| 2 | 212/215 | 282/285 | 227/227 | 283/283 |
| 3 | 215/215 | 282/285 | 227/227 | 283/283 |
| 4 | 212/215 | 282/282 | 227/227 | 265/283 |
| 5 | 215/215 | 282/285 | 227/227 | 283/283 |
| 6 | 215/215 | 282/285 | 227/227 | 265/283 |
| 7 | 215/215 | 282/282 | 227/227 | 265/283 |
| 8 | 212/215 | 282/282 | 227/227 | 265/283 |
| 9 | 212/215 | 282/282 | 227/227 | 265/283 |
| 10 | 212/215 | 282/285 | 227/227 | 283/283 |
| 11 | 212/215 | 282/285 | 227/227 | 283/283 |
| 12 | 212/215 | 282/285 | 227/227 | 265/283 |
|  |  |  |  |  |
| 4-1_May_2017 | | | | |
| 1 | 212/215 | 282/282 | 227/227 | 265/283 |
| 2 | 212/215 | 282/285 | 227/227 | 283/283 |
| 3 | 212/215 | 282/285 | 227/227 | 265/283 |
| 4 | 212/215 | 282/285 | 227/227 | 283/283 |
| 5 | 212/215 | 282/285 | 227/227 | 283/283 |
| 6 | 212/215 | 282/282 | 227/227 | 265/283 |
| 7 | 212/215 | 282/282 | 227/227 | 283/283 |
| 8 | 212/215 | 282/285 | 227/227 | 283/283 |
| 9 | 215/215 | 282/282 | 227/227 | 265/283 |
| 10 | 212/215 | 282/285 | 227/227 | 265/283 |
|  |  |  |  |  |
| 4-4_Nov_2016 | | | | |
| 1 | 215/215 | 282/285 | 227/227 | 283/283 |
| 2 | 215/215 | 282/282 | 227/227 | 265/283 |
| 3 | 215/215 | 282/282 | 227/227 | 283/283 |
| 4 | 215/215 | 282/285 | 227/227 | 283/283 |
| 5 | 215/215 | 282/285 | 227/227 | 265/283 |
| 6 | 212/215 | 282/282 | 227/227 | 265/283 |
| 7 | 215/215 | 282/282 | 227/227 | 265/283 |
| 8 | 215/215 | 282/282 | 227/227 | 283/283 |
| 9 | 215/215 | 282/282 | 227/227 | 283/283 |
| 10 | 215/215 | 282/285 | 227/227 | 283/283 |
| 11 | 215/215 | 282/282 | 227/227 | 283/283 |
| 12 | 215/215 | 282/285 | 227/227 | 283/283 |
| 13 | 212/215 | 282/282 | 227/227 | 265/283 |
| 14 | 212/215 | 282/285 | 227/227 | 283/283 |
| 15 | 215/215 | 282/282 | 227/227 | 265/283 |
| 16 | 215/215 | 282/282 | 227/227 | 283/283 |
|  |  |  |  |  |
| 4-4_May_2017 | | | | |
| 1 | 212/215 | 282/285 | 227/227 | 265/283 |
| 2 | 212/215 | 282/282 | 227/227 | 265/283 |
| 3 | 212/215 | 282/282 | 227/227 | 283/283 |
|  |  |  |  |  |
| 4-4_Aug_2020 | | | | |
| 1 | 212/215 | 282/282 | 227/227 | 283/283 |
| 2 | 212/215 | 282/282 | 227/227 | 283/283 |
| 3 | 212/215 | 282/285 | 227/227 | 283/283 |
| 4 | 215/215 | 282/285 | 227/227 | 283/283 |
| 5 | 215/215 | 282/285 | 227/227 | 265/283 |
| 6 | 215/215 | 282/282 | 227/227 | 265/283 |
|  |  |  |  |  |
| 4-5_Aug_2020 | | | | |
| 1 | 215/215 | 282/282 | 227/227 | 265/283 |
| 2 | 212/215 | 282/285 | 227/227 | 265/283 |
| 3 | 215/215 | 282/282 | 227/227 | 283/283 |
| 4 | 212/215 | 282/282 | 227/227 | 283/283 |
|  |  |  |  |  |
| 4-6_May_2017 | | | | |
| 1 | 212/215 | 282/285 | 227/227 | 265/283 |
| 2 | 212/215 | 282/282 | 227/227 | 283/283 |
| 3 | 212/215 | 282/285 | 227/227 | 283/283 |
| 4 | 212/215 | 282/285 | 227/227 | 283/283 |
| 5 | 212/215 | 282/282 | 227/227 | 283/283 |
| 6 | 212/215 | 282/282 | 227/227 | 265/283 |
|  |  |  |  |  |
| 6-4_Jun_2020 | | | | |
| 1 | 215/215 | 282/282 | 221/227 | 283/283 |
| 2 | 215/218 | 282/282 | 221/227 | 271/283 |
| 3 | 215/218 | 282/282 | 221/227 | 283/283 |
| 4 | 215/218 | 282/282 | 227/227 | 271/283 |
| 5 | 218/218 | 282/282 | 227/227 | 283/283 |
| 6 | 215/215 | 282/282 | 227/227 | 271/271 |
| 7 | 215/218 | 282/282 | 221/227 | 271/283 |
| 8 | 215/218 | 282/282 | 221/227 | 271/283 |
|  |  |  |  |  |
| Stump_Apr_2018 | | | | |
| 1 | 215/218 | 282/282 | 221/227 | 271/283 |
| 2 | 215/218 | 282/282 | 221/227 | 271/283 |
| 3 | 215/215 | 282/282 | 221/227 | 271/271 |
| 4 | 215/215 | 282/282 | 227/227 | 271/271 |
| 5 | 215/215 | 282/282 | 227/227 | 283/283 |
| 6 | 215/218 | 282/282 | 227/227 | 283/283 |
| 7 | 215/215 | 282/282 | 227/227 | 271/283 |
| 8 | 215/215 | 282/282 | 227/227 | 283/283 |
| 9 | 215/215 | 282/282 | 221/227 | 271/283 |
| 10 | 215/215 | 282/282 | 227/227 | 271/271 |
| 11 | 215/218 | 282/282 | 221/227 | 271/283 |
| 12 | 215/215 | 282/282 | 227/227 | 271/283 |
| 13 | 215/218 | 282/282 | 221/227 | 271/271 |
| 14 | 215/218 | 282/282 | 221/227 | 271/283 |
| 15 | 215/218 | 282/282 | 221/227 | 283/283 |
| 16 | 215/215 | 282/282 | 221/227 | 271/283 |
| 17 | 215/215 | 282/282 | 227/227 | 271/283 |
|  |  |  |  |  |
| Stump_Mar_2019 | | | | |
| 1 | 215/215 | 282/282 | 221/227 | 271/283 |
| 2 | 215/215 | 282/282 | 221/227 | 271/283 |
| 3 | 215/215 | 282/282 | 221/227 | 271/283 |
| 4 | 215/215 | 282/282 | 221/227 | 283/283 |
| 5 | 215/215 | 282/282 | 227/227 | 283/283 |
| 6 | 215/215 | 282/282 | 221/227 | 283/283 |
| 7 | 215/215 | 282/282 | 221/227 | 283/283 |
| 8 | 215/215 | 282/282 | 221/227 | 271/271 |
|  |  |  |  |  |
| Stump_May_2020 | | | | |
| 1 | 215/218 | 282/282 | 221/227 | 271/271 |
| 2 | 215/215 | 282/282 | 227/227 | 271/283 |
| 3 | 215/218 | 282/282 | 227/227 | 271/283 |
| 4 | 215/218 | 282/282 | 221/227 | 271/283 |
| 5 | 215/218 | 282/282 | 227/227 | 271/283 |
| 6 | 215/218 | 282/282 | 227/227 | 271/283 |
| 7 | 215/218 | 282/282 | 221/227 | 271/283 |
| 8 | 215/215 | 282/282 | 227/227 | 271/283 |
|  |  |  |  |  |
| Laboratory | | | | |
| 1 | 212/215 | 267/267 | 221/284 | 280/283 |
| 2 | 215/215 | 267/267 | 221/221 | 283/283 |
| 3 | 215/215 | 267/282 | 221/284 | 280/283 |
| 4 | 215/215 | 267/282 | 221/284 | 280/283 |
| 5 | 215/215 | 267/267 | 221/284 | 280/283 |
| 6 | 212/215 | 267/267 | 221/221 | 280/283 |
| 7 | 215/215 | 267/282 | 221/284 | 283/283 |
| 8 | 215/215 | 267/282 | 221/221 | 283/283 |
| 9 | 215/215 | 267/282 | 221/284 | 280/283 |
| 10 | 215/215 | 267/282 | 221/284 | 280/283 |
