# Supplementary material for: Colony Suppression and Possible Colony Elimination of the Subterranean Termites Coptotermes formosanus and Reticulitermes speratus by Discontinuous Soil Treatment Using a Diluent of Fipronil Suspension Concentrate
Source: Insects. 2021 Apr 8;12(4):334. doi: 10.3390/insects12040334 (PMC8068406; doi:10.3390/insects12040334)
Supplement: Supplementary file 1 [file insects-12-00334-s001.zip › TableS2.docx]

**Table S2:** Sizes of alleles detected in cohorts of *Reticulitermes speratus* in Isogi Park.

| Cohort | Alleles (bp) | | | |
| --- | --- | --- | --- | --- |
|  | Rs02 | Rs03 | Rs05 | Rs07 |
| 1-1_Oct_2018 | | | | |
| 1 | 246/256 | 196/196 | 210/212 | 192/192 |
| 2 | 246/256 | 196/196 | 210/212 | 192/192 |
| 3 | 246/256 | 196/196 | 210/210 | 192/192 |
| 4 | 246/256 | 196/196 | 210/212 | 192/192 |
| 5 | 246/256 | 196/196 | 210/210 | 192/192 |
| 6 | 246/256 | 196/196 | 210/212 | 192/192 |
| 7 | 246/256 | 196/196 | 210/212 | 192/192 |
| 8 | 246/256 | 196/196 | 210/210 | 192/192 |
|  |  |  |  |  |
| 1-2_Oct_2018 | | | | |
| 1 | 246/256 | 196/196 | 210/210 | 192/192 |
| 2 | 246/256 | 196/196 | 210/212 | 192/192 |
| 3 | 246/256 | 196/196 | 210/212 | 192/192 |
| 4 | 246/256 | 196/196 | 210/210 | 192/192 |
| 5 | 246/256 | 196/196 | 210/212 | 190/190 |
| 6 | 246/256 | 196/196 | 210/210 | 192/192 |
| 7 | 246/256 | 196/196 | 210/212 | 192/192 |
| 8 | 246/256 | 196/196 | 210/210 | 192/192 |
|  |  |  |  |  |
| 1-5_Oct_2018 | | | | |
| 1 | 246/256 | 196/196 | 210/210 | 192/192 |
| 2 | 246/256 | 196/196 | 210/210 | 190/190 |
| 3 | 246/256 | 196/196 | 210/212 | 190/190 |
| 4 | 246/256 | 196/196 | 210/210 | 190/190 |
| 5 | 246/256 | 196/196 | 210/210 | 192/192 |
| 6 | 246/256 | 196/196 | 210/212 | 192/192 |
| 7 | 246/256 | 196/196 | 210/210 | 192/192 |
|  |  |  |  |  |
| 1-5_Mar_2019 | | | | |
| 1 | 246/256 | 196/198 | 210/212 | 192/192 |
| 2 | 246/256 | 196/198 | 210/210 | 192/192 |
| 3 | 246/256 | 196/198 | 210/212 | 192/192 |
| 4 | 246/256 | 196/198 | 210/212 | 192/192 |
| 5 | 246/256 | 196/198 | 210/210 | 192/192 |
| 6 | 246/256 | 196/198 | 210/210 | 192/192 |
| 7 | 246/256 | 196/198 | 210/212 | 192/192 |
| 8 | 246/256 | 196/198 | 210/210 | 192/192 |
|  |  |  |  |  |
| 1-5_Jun_2019 | | | | |
| 1 | 246/256 | 196/198 | 210/212 | 192/192 |
| 2 | 246/256 | 196/196 | 210/210 | 192/192 |
| 3 | 246/256 | 196/196 | 210/212 | 192/192 |
| 4 | 246/256 | 196/198 | 210/212 | 192/192 |
| 5 | 246/256 | 196/196 | 210/212 | 192/192 |
| 6 | 246/256 | 196/196 | 210/210 | 192/192 |
| 7 | 246/256 | 196/198 | 210/210 | 192/192 |
| 8 | 246/256 | 196/198 | 210/212 | 192/192 |
| 9 | 246/256 | 196/198 | 210/210 | 192/192 |
| 10 | 246/256 | 196/198 | 210/210 | 192/192 |
| 11 | 246/256 | 196/198 | 210/212 | 192/192 |
|  |  |  |  |  |
| 1-6_Oct_2018 | | | | |
| 1 | 246/256 | 196/196 | 210/210 | 190/190 |
| 2 | 246/256 | 196/196 | 210/210 | 192/192 |
| 3 | 246/256 | 196/196 | 210/212 | 192/192 |
| 4 | 246/256 | 196/196 | 210/210 | 192/192 |
| 5 | 246/256 | 196/196 | 210/210 | 190/190 |
| 6 | 246/256 | 196/196 | 210/212 | 192/192 |
| 7 | 246/256 | 196/196 | 210/210 | 190/190 |
| 8 | 246/256 | 196/196 | 210/212 | 190/190 |
|  |  |  |  |  |
| 1-7_Oct_2018 | | | | |
| 1 | 246/256 | 196/196 | 210/210 | 192/192 |
| 2 | 246/256 | 196/196 | 210/210 | 192/192 |
| 3 | 246/256 | 196/196 | 210/212 | 192/192 |
| 4 | 246/256 | 196/196 | 210/212 | 192/192 |
| 5 | 246/256 | 196/196 | 210/210 | 192/192 |
| 6 | 246/256 | 196/196 | 210/210 | 192/192 |
| 7 | 246/256 | 196/196 | 210/210 | 192/192 |
| 8 | 246/256 | 196/196 | 210/212 | 190/190 |
|  |  |  |  |  |
| 1-7_Jun_2019 | | | | |
| 1 | 246/256 | 196/198 | 210/212 | 192/192 |
| 2 | 246/256 | 196/196 | 210/212 | 192/192 |
| 3 | 246/256 | 196/198 | 210/210 | 192/192 |
| 4 | 246/256 | 196/198 | 210/212 | 192/192 |
| 5 | 246/256 | 196/196 | 210/212 | 192/192 |
| 6 | 246/256 | 196/196 | 210/212 | 192/192 |
| 7 | 246/256 | 196/198 | 210/212 | 190/190 |
|  |  |  |  |  |
| 5-1_May_2020 | | | | |
| 1 | 252/256 | 196/198 | 210/210 | 192/192 |
| 2 | 246/252 | 196/196 | 210/210 | 192/192 |
| 3 | 246/252 | 196/196 | 210/210 | 192/192 |
| 4 | 256/256 | 196/196 | 210/210 | 192/192 |
| 5 | 246/256 | 196/196 | 210/210 | 192/192 |
| 6 | 246/252 | 196/196 | 210/210 | 192/192 |
| 7 | 256/256 | 196/196 | 210/210 | 192/192 |
| 8 | 246/252 | 196/196 | 210/210 | 192/192 |
|  |  |  |  |  |
| 5-2_May_2020 | | | | |
| 1 | 246/252 | 196/196 | 210/210 | 192/192 |
| 2 | 252/256 | 196/196 | 210/210 | 192/192 |
| 3 | 246/252 | 196/196 | 210/210 | 192/192 |
| 4 | 252/256 | 196/196 | 210/210 | 192/192 |
| 5 | 252/256 | 196/196 | 210/210 | 192/192 |
| 6 | 252/256 | 196/196 | 210/210 | 192/192 |
| 7 | 246/252 | 196/196 | 210/210 | 192/192 |
| 8 | 246/252 | 196/196 | 210/210 | 192/192 |
| 9 | 246/252 | 196/196 | 210/210 | 192/192 |
|  |  |  |  |  |
| 5-3_May_2020 | | | | |
| 1 | 252/256 | 196/196 | 210/210 | 192/192 |
| 2 | 246/252 | 196/196 | 210/210 | 192/192 |
| 3 | 246/252 | 196/196 | 210/210 | 192/192 |
| 4 | 256/256 | 196/196 | 210/210 | 192/192 |
| 5 | 252/256 | 196/196 | 210/210 | 192/192 |
| 6 | 252/256 | 196/196 | 210/210 | 192/192 |
| 7 | 246/252 | 196/196 | 210/210 | 192/192 |
| 8 | 252/256 | 196/196 | 210/210 | 192/192 |
|  |  |  |  |  |
| 6-2_May_2020 | | | | |
| 1 | 246/246 | 196/196 | 210/210 | 192/192 |
| 2 | 246/246 | 196/196 | 210/210 | 192/192 |
| 3 | 246/246 | 196/196 | 210/210 | 192/192 |
| 4 | 248/252 | 196/196 | 210/210 | 192/192 |
| 5 | 246/246 | 196/196 | 210/210 | 192/192 |
| 6 | 248/248 | 196/196 | 210/210 | 192/192 |
| 7 | 248/252 | 196/196 | 210/210 | 192/192 |
| 8 | 248/248 | 196/196 | 210/210 | 192/192 |
|  |  |  |  |  |
| 6-2_Sep_2020 | | | | |
| 1 | 248/252 | 196/196 | 210/210 | 192/192 |
| 2 | 248/252 | 196/196 | 210/210 | 192/192 |
| 3 | 246/248 | 196/196 | 210/210 | 192/192 |
| 4 | 246/252 | 196/196 | 210/210 | 192/192 |
| 5 | 246/246 | 196/196 | 210/210 | 192/192 |
| 6 | 248/252 | 196/196 | 210/210 | 192/192 |
| 7 | 246/246 | 196/196 | 210/210 | 192/192 |
| 8 | 248/252 | 196/196 | 210/210 | 192/192 |
|  |  |  |  |  |
| 6-3_Sep_2020 | | | | |
| 1 | 246/246 | 196/196 | 210/210 | 192/192 |
| 2 | 246/248 | 196/196 | 210/210 | 192/192 |
| 3 | 246/248 | 196/196 | 210/210 | 192/192 |
| 4 | 246/246 | 196/196 | 210/210 | 192/192 |
| 5 | 246/248 | 196/196 | 210/210 | 192/192 |
| 6 | 246/252 | 196/196 | 210/210 | 192/192 |
| 7 | 246/252 | 196/196 | 210/210 | 192/192 |
| 8 | 246/246 | 196/196 | 210/210 | 192/192 |
|  |  |  |  |  |
| Laboratory | | | | |
| 1 | 236/256 | 198/198 | 208/212 | 192/192 |
| 2 | 248/248 | 194/198 | 208/212 | 190/190 |
| 3 | 236/256 | 192/196 | 208/210 | 192/192 |
| 4 | 236/256 | 194/198 | 208/212 | 192/192 |
| 5 | 236/256 | 196/198 | 208/210 | 192/192 |
| 6 | 248/248 | 194/198 | 208/212 | 192/192 |
| 7 | 236/256 | 192/196 | 208/210 | 192/192 |
| 8 | 236/256 | 192/196 | 208/210 | 192/192 |
| 9 | 236/256 | 192/196 | 208/210 | 192/192 |
| 10 | 248/256 | 196/198 | 210/210 | 192/192 |
| 11 | 248/248 | 194/196 | 208/212 | 192/192 |
| 12 | 236/256 | 196/198 | 208/212 | 192/192 |
