# Supplementary material for: Colony Suppression and Possible Colony Elimination of the Subterranean Termites Coptotermes formosanus and Reticulitermes speratus by Discontinuous Soil Treatment Using a Diluent of Fipronil Suspension Concentrate
Source: Insects. 2021 Apr 8;12(4):334. doi: 10.3390/insects12040334 (PMC8068406; doi:10.3390/insects12040334)
Supplement: Supplementary file 1 [file insects-12-00334-s001.zip › TableS3.docx]

**Table S3:** Sizes of alleles detected in cohorts of *Reticulitermes speratus* at Kindai University.

| Cohort | Alleles (bp) | | | |
| --- | --- | --- | --- | --- |
|  | Rs02 | Rs03 | Rs05 | Rs07 |
| 1_Oct_2015 | | | | |
| 1 | 246/248 | 196/200 | 210/210 | 192/192 |
| 2 | 246/248 | 192/200 | 210/210 | 192/192 |
| 3 | 246/246 | 192/200 | 210/210 | 192/192 |
| 4 | 246/248 | 192/200 | 210/210 | 192/192 |
| 5 | 246/248 | 192/200 | 210/210 | 192/192 |
| 6 | 246/248 | 196/200 | 210/210 | 192/192 |
| 7 | 246/248 | 196/200 | 210/210 | 192/192 |
| 8 | 246/248 | 196/200 | 210/210 | 192/192 |
| 9 | 246/248 | 196/196 | 210/210 | 192/192 |
|  |  |  |  |  |
| 1_Nov_2018 | | | | |
| 1 | 248/248 | 196/198 | 210/210 | 192/192 |
| 2 | 248/248 | 196/198 | 210/210 | 192/192 |
| 3 | 248/248 | 198/198 | 210/210 | 192/192 |
| 4 | 248/248 | 198/198 | 210/210 | 192/192 |
| 5 | 248/248 | 198/198 | 210/210 | 192/192 |
| 6 | 248/248 | 198/198 | 210/210 | 192/192 |
| 7 | 248/248 | 196/198 | 210/210 | 192/192 |
| 8 | 248/248 | 196/198 | 210/210 | 192/192 |
|  |  |  |  |  |
| 1_Dec_2018 | | | | |
| 1 | 248/248 | 196/198 | 210/210 | 192/192 |
| 2 | 248/248 | 196/198 | 210/210 | 192/192 |
| 3 | 248/248 | 196/198 | 210/210 | 192/192 |
| 4 | 248/248 | 196/198 | 210/210 | 192/192 |
| 5 | 248/248 | 198/198 | 210/210 | 192/192 |
| 6 | 248/248 | 196/196 | 210/210 | 192/192 |
| 7 | 248/248 | 196/198 | 210/210 | 192/192 |
| 8 | 248/248 | 198/198 | 210/210 | 192/192 |
|  |  |  |  |  |
| 1_May_2019 | | | | |
| 1 | 248/248 | 198/198 | 210/210 | 192/192 |
| 2 | 248/248 | 196/198 | 210/210 | 192/192 |
| 3 | 248/248 | 198/198 | 210/210 | 192/192 |
| 4 | 248/248 | 198/198 | 210/210 | 192/192 |
| 5 | 248/248 | 196/198 | 210/210 | 192/192 |
| 6 | 248/248 | 198/198 | 210/210 | 192/192 |
| 7 | 248/248 | 198/198 | 210/210 | 192/192 |
| 8 | 248/248 | 198/198 | 210/210 | 192/192 |
| 9 | 248/248 | 198/198 | 210/210 | 192/192 |
| 10 | 248/248 | 198/198 | 210/210 | 192/192 |
| 11 | 248/248 | 198/198 | 210/210 | 192/192 |
| 12 | 248/248 | 198/198 | 210/210 | 192/192 |
|  |  |  |  |  |
| 1_Nov_2019 | | | | |
| 1 | 248/248 | 196/198 | 210/210 | 192/192 |
| 2 | 248/248 | 196/198 | 210/210 | 192/192 |
| 3 | 248/256 | 198/198 | 210/210 | 192/192 |
| 4 | 248/252 | 198/198 | 210/210 | 192/192 |
| 5 | 248/256 | 198/198 | 210/210 | 192/192 |
| 6 | 248/248 | 198/198 | 210/210 | 192/192 |
| 7 | 248/252 | 196/198 | 210/210 | 192/192 |
| 8 | 248/256 | 198/198 | 210/210 | 192/192 |
| 9 | 248/252 | 196/198 | 210/210 | 192/192 |
| 10 | 248/256 | 198/198 | 210/210 | 192/192 |
| 11 | 248/248 | 198/198 | 210/210 | 192/192 |
|  |  |  |  |  |
| 2_Dec_2014 | | | | |
| 1 | 246/246 | 198/202 | 210/210 | 190/192 |
| 2 | 248/248 | 198/202 | 210/210 | 190/192 |
| 3 | 248/248 | 194/202 | 210/210 | 190/192 |
| 4 | 248/248 | 194/202 | 210/210 | 190/192 |
| 5 | 246/246 | 194/198 | 210/210 | 190/192 |
| 6 | 248/248 | 194/202 | 210/210 | 190/192 |
| 7 | 246/252 | 194/194 | 210/210 | 190/192 |
| 8 | 248/248 | 194/198 | 210/210 | 190/192 |
| 9 | 246/246 | 198/202 | 210/210 | 190/192 |
| 10 | 248/248 | 198/198 | 210/210 | 190/192 |
| 11 | 248/248 | 194/202 | 210/210 | 190/192 |
| 12 | 246/246 | 198/198 | 210/210 | 190/192 |
|  |  |  |  |  |
| 2_Nov_2019 | | | | |
| 1 | 248/248 | 196/198 | 210/210 | 192/192 |
| 2 | 248/248 | 196/198 | 210/210 | 192/192 |
| 3 | 248/248 | 198/198 | 210/210 | 192/192 |
| 4 | 246/248 | 198/198 | 210/210 | 192/192 |
| 5 | 248/248 | 196/198 | 210/210 | 192/192 |
| 6 | 248/248 | 198/198 | 210/210 | 192/192 |
| 7 | 248/248 | 196/198 | 210/210 | 192/192 |
| 8 | 248/248 | 198/198 | 210/210 | 192/192 |
| 9 | 248/248 | 196/198 | 210/210 | 192/192 |
| 10 | 248/248 | 198/198 | 210/210 | 192/192 |
| 11 | 248/248 | 198/198 | 210/210 | 192/192 |
| 12 | 248/248 | 196/198 | 210/210 | 192/192 |
|  |  |  |  |  |
| 3_Nov_2018 | | | | |
| 1 | 248/248 | 196/198 | 210/210 | 192/192 |
| 2 | 248/248 | 196/198 | 210/210 | 192/192 |
| 3 | 248/248 | 196/198 | 210/210 | 192/192 |
| 4 | 248/248 | 196/198 | 210/210 | 192/192 |
| 5 | 248/248 | 198/198 | 210/210 | 192/192 |
| 6 | 248/248 | 198/198 | 210/210 | 192/192 |
| 7 | 248/248 | 196/198 | 210/210 | 192/192 |
| 8 | 248/248 | 196/198 | 210/210 | 192/192 |
|  |  |  |  |  |
| 3_Dec_2018 | | | | |
| 1 | 248/248 | 198/198 | 210/210 | 192/192 |
| 2 | 248/248 | 196/198 | 210/210 | 192/192 |
| 3 | 248/248 | 196/198 | 210/210 | 192/192 |
| 4 | 248/248 | 196/196 | 210/210 | 192/192 |
| 5 | 248/248 | 198/198 | 210/210 | 192/192 |
| 6 | 248/248 | 196/198 | 210/210 | 192/192 |
| 7 | 248/248 | 196/198 | 210/210 | 192/192 |
| 8 | 248/248 | 196/198 | 210/210 | 192/192 |
|  |  |  |  |  |
| 3_May_2019 | | | | |
| 1 | 248/248 | 196/198 | 210/210 | 192/192 |
| 2 | 246/248 | 198/198 | 210/210 | 192/192 |
| 3 | 248/248 | 198/198 | 210/210 | 192/192 |
| 4 | 248/248 | 198/198 | 210/210 | 192/192 |
| 5 | 248/248 | 198/198 | 210/210 | 192/192 |
| 6 | 248/248 | 196/198 | 210/210 | 192/192 |
| 7 | 246/248 | 196/198 | 210/210 | 192/192 |
| 8 | 248/248 | 198/198 | 210/210 | 192/192 |
| 9 | 248/248 | 196/198 | 210/210 | 192/192 |
| 10 | 248/248 | 198/198 | 210/210 | 192/192 |
| 11 | 248/248 | 196/198 | 210/210 | 192/192 |
| 12 | 248/248 | 198/198 | 210/210 | 192/192 |
|  |  |  |  |  |
| 4_Oct_2015 | | | | |
| 1 | 246/248 | 198/198 | 210/210 | 192/192 |
| 2 | 256/256 | 198/198 | 210/210 | 192/192 |
| 3 | 246/248 | 198/198 | 210/210 | 192/192 |
| 4 | 256/256 | 198/198 | 210/210 | 192/192 |
| 5 | 248/256 | 198/198 | 210/210 | 192/192 |
| 6 | 246/256 | 198/198 | 210/214 | 192/192 |
| 7 | 248/256 | 198/200 | 214/214 | 192/192 |
| 8 | 246/256 | 196/198 | 210/214 | 192/192 |
| 9 | 246/248 | 196/198 | 210/210 | 192/192 |
| 10 | 248/256 | 198/198 | 210/214 | 192/192 |
| 11 | 256/256 | 198/198 | 210/214 | 192/192 |
| 12 | 246/248 | 198/198 | 210/210 | 192/192 |
| 13 | 248/256 | 198/198 | 210/210 | 192/192 |
| 14 | 246/248 | 198/198 | 210/210 | 192/192 |
|  |  |  |  |  |
| 5_Dec_2014 | | | | |
| 1 | 246/248 | 196/200 | 210/210 | 192/192 |
| 2 | 246/248 | 196/196 | 210/210 | 192/192 |
| 3 | 248/248 | 196/196 | 210/210 | 192/192 |
| 4 | 248/248 | 196/196 | 210/210 | 192/192 |
| 5 | 248/248 | 192/200 | 210/210 | 192/192 |
| 6 | 248/248 | 196/196 | 210/210 | 192/192 |
| 7 | 246/246 | 192/196 | 210/210 | 192/192 |
| 8 | 246/246 | 196/196 | 210/210 | 192/192 |
| 9 | 246/248 | 196/200 | 210/210 | 192/192 |
| 10 | 246/248 | 192/196 | 210/210 | 192/192 |
| 11 | 246/248 | 196/200 | 210/210 | 192/192 |
| 12 | 246/248 | 192/196 | 210/210 | 192/192 |
|  |  |  |  |  |
| 5_Oct_2015 | | | | |
| 1 | 246/248 | 192/200 | 210/210 | 192/192 |
| 2 | 246/248 | 196/200 | 210/210 | 192/192 |
| 3 | 246/246 | 196/200 | 210/210 | 192/192 |
| 4 | 246/248 | 192/196 | 210/210 | 192/192 |
| 5 | 246/248 | 196/196 | 210/210 | 192/192 |
| 6 | 246/248 | 196/200 | 210/210 | 192/192 |
| 7 | 246/248 | 192/196 | 210/210 | 192/192 |
| 8 | 246/246 | 196/200 | 210/210 | 192/192 |
| 9 | 248/248 | 196/200 | 210/210 | 192/192 |
| 10 | 246/246 | 192/200 | 210/210 | 192/192 |
| 11 | 246/248 | 196/196 | 210/210 | 192/192 |
| 12 | 246/248 | 192/196 | 210/210 | 192/192 |
| 13 | 248/248 | 192/200 | 210/210 | 192/192 |
| 14 | 246/246 | 192/200 | 210/210 | 192/192 |
| 15 | 246/248 | 196/200 | 210/210 | 192/192 |
| 16 | 246/248 | 196/200 | 210/210 | 192/192 |
| 17 | 246/248 | 196/200 | 210/210 | 192/192 |
|  |  |  |  |  |
| 5_Nov_2018 | | | | |
| 1 | 248/248 | 196/198 | 210/210 | 192/192 |
| 2 | 248/248 | 196/198 | 210/210 | 192/192 |
| 3 | 248/248 | 198/198 | 210/210 | 192/192 |
| 4 | 248/248 | 198/198 | 210/210 | 192/192 |
| 5 | 248/248 | 196/198 | 210/210 | 192/192 |
| 6 | 248/248 | 196/198 | 210/210 | 192/192 |
| 7 | 248/248 | 196/198 | 210/210 | 192/192 |
| 8 | 248/248 | 196/198 | 210/210 | 192/192 |
|  |  |  |  |  |
| 6_Oct_2015 | | | | |
| 1 | 246/246 | 196/200 | 210/210 | 192/192 |
| 2 | 246/246 | 196/196 | 210/210 | 192/192 |
| 3 | 246/246 | 196/196 | 210/210 | 192/192 |
| 4 | 246/246 | 196/200 | 210/210 | 192/192 |
| 5 | 246/246 | 196/196 | 210/210 | 192/192 |
| 6 | 246/248 | 196/196 | 210/210 | 192/192 |
| 7 | 246/248 | 196/200 | 210/210 | 192/192 |
| 8 | 246/248 | 196/196 | 210/210 | 192/192 |
| 9 | 246/248 | 196/200 | 210/210 | 192/192 |
| 10 | 246/246 | 192/200 | 210/210 | 192/192 |
| 11 | 246/246 | 196/200 | 210/210 | 192/192 |
| 12 | 246/248 | 196/200 | 210/210 | 192/192 |
| 13 | 246/246 | 192/200 | 210/210 | 192/192 |
| 14 | 246/246 | 196/200 | 210/210 | 192/192 |
| 15 | 246/246 | 196/200 | 210/210 | 192/192 |
| 16 | 246/246 | 196/196 | 210/210 | 192/192 |
| 17 | 246/248 | 196/196 | 210/210 | 192/192 |
| 18 | 246/248 | 196/200 | 210/210 | 192/192 |
| 19 | 246/248 | 196/196 | 210/210 | 192/192 |
|  |  |  |  |  |
| 6_Nov_2019 | | | | |
| 1 | 246/248 | 198/198 | 210/210 | 192/192 |
| 2 | 246/248 | 198/198 | 210/210 | 192/192 |
| 3 | 248/248 | 198/198 | 210/210 | 192/192 |
| 4 | 246/248 | 198/198 | 210/210 | 192/192 |
| 5 | 248/248 | 198/198 | 210/210 | 192/192 |
| 6 | 246/248 | 196/198 | 210/210 | 192/192 |
| 7 | 246/248 | 198/198 | 210/210 | 192/192 |
| 8 | 246/248 | 196/198 | 210/210 | 192/192 |
| 9 | 248/248 | 198/198 | 210/210 | 192/192 |
| 10 | 248/248 | 198/198 | 210/210 | 192/192 |
|  |  |  |  |  |
| 7_Oct_2015 | | | | |
| 1 | 248/248 | 196/198 | 210/210 | 192/192 |
| 2 | 248/248 | 196/198 | 210/214 | 192/192 |
| 3 | 246/248 | 198/198 | 210/214 | 192/192 |
| 4 | 250/250 | 198/200 | 214/218 | 194/194 |
|  |  |  |  |  |
| 7_Nov_2017 | | | | |
| 1 | 248/248 | 198/198 | 210/210 | 192/192 |
| 2 | 248/248 | 198/198 | 210/210 | 192/192 |
| 3 | 246/248 | 198/198 | 210/210 | 192/192 |
| 4 | 246/248 | 198/198 | 210/210 | 192/192 |
| 5 | 248/248 | 198/198 | 210/210 | 192/192 |
| 6 | 248/248 | 198/198 | 210/210 | 192/192 |
| 7 | 246/248 | 198/198 | 210/210 | 192/192 |
| 8 | 246/248 | 196/198 | 210/210 | 192/192 |
| 9 | 248/248 | 198/198 | 210/210 | 192/192 |
| 10 | 248/248 | 198/198 | 210/210 | 192/192 |
| 11 | 248/248 | 198/198 | 210/210 | 192/192 |
| 12 | 248/248 | 198/198 | 210/210 | 192/192 |
|  |  |  |  |  |
| 8_Nov_2019 | | | | |
| 1 | 248/256 | 198/202 | 210/214 | 192/192 |
| 2 | 256/256 | 198/202 | 210/210 | 192/192 |
| 3 | 256/256 | 198/202 | 210/210 | 192/192 |
| 4 | 256/256 | 198/202 | 210/214 | 192/192 |
| 5 | 256/256 | 198/202 | 210/210 | 192/192 |
| 6 | 248/256 | 198/198 | 210/214 | 192/192 |
| 7 | 256/256 | 198/198 | 210/214 | 192/192 |
| 8 | 256/256 | 198/198 | 210/210 | 192/192 |
|  |  |  |  |  |
| 9_May_2016 | | | | |
| 1 | 246/248 | 198/198 | 210/210 | 192/192 |
| 2 | 248/248 | 198/198 | 210/210 | 192/192 |
| 3 | 248/248 | 198/198 | 210/210 | 192/192 |
| 4 | 248/248 | 196/198 | 210/210 | 192/192 |
| 5 | 248/248 | 198/198 | 210/210 | 192/192 |
| 6 | 248/248 | 196/198 | 210/210 | 192/192 |
| 7 | 248/248 | 196/198 | 210/210 | 192/192 |
| 8 | 248/248 | 198/198 | 210/210 | 192/192 |
| 9 | 248/248 | 198/198 | 210/210 | 192/192 |
| 10 | 248/248 | 198/198 | 210/210 | 192/192 |
| 11 | 248/248 | 198/198 | 210/210 | 192/192 |
| 12 | 248/248 | 196/198 | 210/210 | 192/192 |
|  |  |  |  |  |
| 11_May_2019 | | | | |
| 1 | 248/248 | 196/198 | 210/214 | 192/192 |
| 2 | 248/248 | 198/198 | 210/210 | 192/192 |
| 3 | 246/256 | 198/198 | 210/214 | 192/192 |
| 4 | 246/248 | 198/198 | 210/210 | 192/192 |
| 5 | 246/248 | 196/198 | 210/214 | 192/192 |
| 6 | 246/248 | 198/198 | 210/214 | 192/192 |
| 7 | 248/248 | 196/198 | 210/214 | 192/192 |
| 8 | 246/248 | 196/198 | 210/214 | 192/192 |
| 9 | 248/248 | 196/198 | 210/210 | 192/192 |
| 10 | 248/248 | 198/198 | 210/210 | 192/192 |
| 11 | 248/248 | 198/198 | 210/214 | 192/192 |
|  |  |  |  |  |
| 12_May_2016 | | | | |
| 1 | 244/248 | 196/198 | 210/210 | 190/192 |
| 2 | 244/244 | 196/198 | 210/210 | 190/192 |
| 3 | 244/244 | 196/196 | 210/210 | 190/190 |
| 4 | 244/244 | 196/196 | 210/210 | 190/190 |
| 5 | 248/248 | 196/198 | 210/210 | 190/192 |
| 6 | 248/248 | 196/198 | 210/210 | 190/192 |
| 7 | 248/248 | 196/198 | 210/210 | 190/192 |
| 8 | 248/248 | 196/198 | 210/210 | 190/192 |
| 9 | 248/248 | 196/198 | 210/210 | 190/192 |
| 10 | 248/248 | 196/198 | 210/210 | 190/190 |
| 11 | 244/244 | 198/198 | 210/210 | 190/192 |
| 12 | 248/248 | 196/198 | 210/210 | 190/192 |
|  |  |  |  |  |
| 13_Dec_2014 | | | | |
| 1 | 248/248 | 198/202 | 210/210 | 190/192 |
| 2 | 248/248 | 198/202 | 210/210 | 190/192 |
| 3 | 248/248 | 198/198 | 210/210 | 190/192 |
| 4 | 248/252 | 194/194 | 210/210 | 190/192 |
| 5 | 248/248 | 198/198 | 210/210 | 190/192 |
| 6 | 248/248 | 202/202 | 210/210 | 190/192 |
|  |  |  |  |  |
| 13_Oct_2015 | | | | |
| 1 | 248/248 | 198/202 | 210/210 | 192/192 |
| 2 | 248/248 | 198/198 | 210/210 | 192/192 |
| 3 | 248/248 | 198/202 | 210/210 | 192/192 |
| 4 | 248/248 | 198/202 | 210/210 | 192/192 |
| 5 | 248/248 | 198/202 | 210/210 | 190/192 |
| 6 | 248/256 | 198/198 | 210/210 | 192/192 |
| 7 | 247/248 | 198/202 | 210/210 | 192/192 |
| 8 | 248/248 | 198/202 | 210/210 | 192/192 |
| 9 | 248/248 | 198/202 | 210/210 | 192/192 |
| 10 | 252/248 | 198/202 | 210/210 | 190/192 |
| 11 | 248/248 | 198/202 | 210/210 | 192/192 |
| 12 | 248/248 | 198/202 | 210/210 | 192/192 |
| 13 | 248/248 | 198/198 | 210/210 | 190/190 |
| 14 | 248/248 | 198/198 | 210/210 | 190/190 |
| 15 | 248/248 | 198/198 | 210/210 | 190/190 |
| 16 | 248/248 | 198/198 | 210/210 | 190/192 |
| 17 | 248/248 | 198/202 | 210/210 | 192/192 |
| 18 | 248/248 | 198/202 | 210/210 | 192/192 |
|  |  |  |  |  |
| 14_May_2019 | | | | |
| 1 | 248/248 | 196/198 | 210/210 | 192/192 |
| 2 | 246/248 | 196/198 | 210/214 | 192/192 |
| 3 | 256/256 | 196/198 | 210/214 | 192/192 |
| 4 | 246/256 | 198/198 | 210/214 | 192/192 |
| 5 | 248/248 | 196/198 | 210/210 | 192/192 |
| 6 | 256/256 | 196/198 | 210/214 | 192/192 |
| 7 | 248/248 | 196/198 | 210/210 | 192/192 |
| 8 | 246/256 | 196/198 | 210/210 | 192/192 |
| 9 | 248/248 | 196/198 | 210/214 | 192/192 |
| 10 | 246/248 | 198/198 | 210/210 | 192/192 |
| 11 | 248/248 | 198/198 | 210/210 | 192/192 |
| 12 | 248/248 | 196/198 | 210/214 | 192/192 |
|  |  |  |  |  |
| 15_Dec_2018 | | | | |
| 1 | 248/248 | 196/198 | 210/214 | 192/192 |
| 2 | 248/248 | 196/198 | 210/210 | 192/192 |
| 3 | 248/256 | 196/198 | 210/214 | 192/192 |
| 4 | 256/256 | 196/198 | 210/210 | 192/192 |
| 5 | 246/256 | 196/196 | 210/210 | 192/192 |
| 6 | 248/248 | 196/198 | 210/214 | 192/192 |
| 7 | 248/248 | 196/198 | 210/214 | 192/192 |
| 8 | 256/256 | 196/198 | 210/214 | 192/192 |
|  |  |  |  |  |
| 16_Sept_2013 | | | | |
| 1 | 248/256 | 202/202 | 210/210 | 192/192 |
| 2 | 248/248 | 196/202 | 210/210 | 192/192 |
| 3 | 248/256 | 196/202 | 210/210 | 192/192 |
| 4 | 248/256 | 196/198 | 210/210 | 192/192 |
| 5 | 248/248 | 196/198 | 210/210 | 192/192 |
| 6 | 256/256 | 196/198 | 210/210 | 192/192 |
| 7 | 256/256 | 196/202 | 210/210 | 192/192 |
| 8 | 248/248 | 196/198 | 210/210 | 192/192 |
| 9 | 256/256 | 196/198 | 210/210 | 192/192 |
| 10 | 256/256 | 196/198 | 210/210 | 192/192 |
| 11 | 256/256 | 196/198 | 210/210 | 192/192 |
| 12 | 248/256 | 202/202 | 210/210 | 192/192 |
| 13 | 248/248 | 196/202 | 210/210 | 192/192 |
| 14 | 248/256 | 202/202 | 210/210 | 192/192 |
|  |  |  |  |  |
| 16_Dec_2018 | | | | |
| 1 | 248/248 | 196/198 | 210/214 | 192/192 |
| 2 | 248/248 | 196/198 | 210/210 | 192/192 |
| 3 | 248/248 | 196/198 | 210/210 | 192/192 |
| 4 | 256/256 | 196/198 | 210/210 | 192/192 |
| 5 | 248/248 | 196/198 | 210/210 | 192/192 |
| 6 | 246/256 | 196/198 | 210/210 | 192/192 |
| 7 | 248/248 | 196/198 | 210/210 | 192/192 |
| 8 | 246/248 | 196/196 | 210/210 | 192/192 |
|  |  |  |  |  |
| 16_May_2019 | | | | |
| 1 | 256/256 | 196/198 | 210/210 | 192/192 |
| 2 | 248/248 | 196/198 | 210/214 | 192/192 |
| 3 | 248/248 | 196/198 | 210/210 | 192/192 |
| 4 | 246/256 | 198/198 | 210/210 | 192/192 |
| 5 | 248/248 | 196/198 | 210/214 | 192/192 |
| 6 | 246/248 | 198/198 | 210/214 | 192/192 |
| 7 | 246/256 | 198/198 | 210/210 | 192/192 |
| 8 | 248/248 | 196/198 | 210/210 | 192/192 |
| 9 | 256/256 | 196/198 | 210/210 | 192/192 |
| 10 | 248/248 | 198/198 | 210/210 | 192/192 |
| 11 | 248/248 | 198/198 | 210/214 | 192/192 |
|  |  |  |  |  |
| 18_Aug_2018 | | | | |
| 1 | 248/248 | 198/198 | 210/214 | 192/192 |
| 2 | 256/256 | 198/198 | 210/214 | 192/192 |
| 3 | 248/256 | 196/198 | 210/210 | 192/192 |
| 4 | 256/256 | 198/198 | 210/210 | 192/192 |
| 5 | 246/248 | 198/198 | 210/210 | 192/192 |
| 6 | 248/256 | 198/198 | 210/214 | 192/192 |
| 7 | 248/248 | 196/198 | 210/210 | 192/192 |
| 8 | 248/256 | 196/198 | 210/210 | 192/192 |
| 9 | 246/256 | 198/198 | 210/214 | 192/192 |
| 10 | 246/248 | 196/198 | 210/210 | 192/192 |
| 11 | 248/248 | 196/198 | 210/214 | 192/192 |
|  |  |  |  |  |
| 18_Dec_2018 | | | | |
| 1 | 246/256 | 196/198 | 210/210 | 188/192 |
| 2 | 246/256 | 196/198 | 210/210 | 188/192 |
| 3 | 246/256 | 196/198 | 210/210 | 188/192 |
| 4 | 246/256 | 196/198 | 210/210 | 188/192 |
| 5 | 254/254 | 196/198 | 210/210 | 188/192 |
| 6 | 246/246 | 196/198 | 210/210 | 188/192 |
| 7 | 256/256 | 196/198 | 210/210 | 192/192 |
| 8 | 254/254 | 196/198 | 210/210 | 192/192 |
|  |  |  |  |  |
| 18_Nov_2019 | | | | |
| 1 | 246/256 | 198/198 | 210/210 | 192/192 |
| 2 | 256/256 | 198/198 | 210/210 | 192/192 |
| 3 | 246/254 | 198/198 | 210/210 | 192/192 |
| 4 | 246/256 | 198/198 | 210/210 | 192/192 |
| 5 | 246/256 | 198/198 | 210/210 | 192/192 |
| 6 | 256/256 | 198/198 | 210/210 | 192/192 |
| 7 | 254/256 | 198/198 | 210/210 | 192/192 |
| 8 | 246/256 | 198/198 | 210/210 | 192/192 |
| 9 | 256/256 | 198/198 | 210/210 | 192/192 |
| 10 | 256/256 | 198/198 | 210/210 | 192/192 |
| 11 | 256/256 | 198/198 | 210/210 | 192/192 |
|  |  |  |  |  |
| 20_Aug_2018 | | | | |
| 1 | 254/254 | 198/198 | 210/210 | 188/188 |
| 2 | 256/256 | 198/198 | 210/210 | 192/192 |
| 3 | 246/254 | 198/198 | 210/210 | 188/192 |
| 4 | 246/256 | 198/198 | 210/210 | 188/192 |
| 5 | 254/256 | 198/198 | 210/210 | 192/192 |
| 6 | 256/256 | 198/198 | 210/210 | 192/192 |
| 7 | 256/256 | 198/198 | 210/210 | 192/192 |
| 8 | 246/256 | 198/198 | 210/210 | 188/192 |
| 9 | 256/256 | 198/198 | 210/210 | 192/192 |
| 10 | 254/254 | 198/198 | 210/210 | 192/192 |
|  |  |  |  |  |
| 27_Aug_2018 | | | | |
| 1 | 248/256 | 196/198 | 210/214 | 192/192 |
| 2 | 256/256 | 196/198 | 210/210 | 192/192 |
| 3 | 246/248 | 196/198 | 210/210 | 192/192 |
| 4 | 248/256 | 196/198 | 210/210 | 192/192 |
| 5 | 246/256 | 196/198 | 210/210 | 192/192 |
| 6 | 246/256 | 196/198 | 210/214 | 192/192 |
| 7 | 248/256 | 196/198 | 210/210 | 192/192 |
| 8 | 248/256 | 196/198 | 210/210 | 192/192 |
| 9 | 248/256 | 196/198 | 210/210 | 192/192 |
| 10 | 248/256 | 196/198 | 210/210 | 192/192 |
| 11 | 248/256 | 196/198 | 210/210 | 192/192 |
|  |  |  |  |  |
| Laboratory | | | | |
| 1 | 236/256 | 198/198 | 208/212 | 192/192 |
| 2 | 248/248 | 194/198 | 208/212 | 190/190 |
| 3 | 236/256 | 192/196 | 208/210 | 192/192 |
| 4 | 236/256 | 194/198 | 208/212 | 192/192 |
| 5 | 236/256 | 196/198 | 208/210 | 192/192 |
| 6 | 248/248 | 194/198 | 208/212 | 192/192 |
| 7 | 236/256 | 192/196 | 208/210 | 192/192 |
| 8 | 236/256 | 192/196 | 208/210 | 192/192 |
| 9 | 236/256 | 192/196 | 208/210 | 192/192 |
| 10 | 248/256 | 196/198 | 210/210 | 192/192 |
| 11 | 248/248 | 194/196 | 208/212 | 192/192 |
| 12 | 236/256 | 196/198 | 208/212 | 192/192 |
