# Supplementary material for: Colony Suppression and Possible Colony Elimination of the Subterranean Termites Coptotermes formosanus and Reticulitermes speratus by Discontinuous Soil Treatment Using a Diluent of Fipronil Suspension Concentrate
Source: Insects. 2021 Apr 8;12(4):334. doi: 10.3390/insects12040334 (PMC8068406; doi:10.3390/insects12040334)
Supplement: Supplementary file 1 [file insects-12-00334-s001.zip › TableS4.docx]

**Table S4:** Allele frequency in each locus of *Coptotermes formosanus* in Isogi Park.

| **Locus: Cf4-4** | Population | No of Alleles | Alleles (bp) | | | | | | |
| --- | --- | --- | --- | --- | --- | --- | --- | --- | --- |
| Cohort |  |  | 212 | | 215 | | 218 | | Total |
| 1-1_Nov_2016 | A | 1 | 0 | | 20 | | 0 | | 20 |
| 1-1_May_2017 | A | 2 | 0 | | 19 | | 3 | | 22 |
| 1-6_Oct_2019 | A | 2 | 0 | | 18 | | 2 | | 20 |
| 2-2_May_2017 | B | 2 | 1 | | 11 | | 0 | | 12 |
| 3-1_May_2017 | B | 2 | 5 | | 11 | | 0 | | 16 |
| 3-1_Oct_2019 | B | 2 | 5 | | 15 | | 0 | | 20 |
| 3-2_Oct_2019 | B | 2 | 3 | | 17 | | 0 | | 20 |
| 4-1_Nov_2016 | B | 2 | 7 | | 17 | | 0 | | 24 |
| 4-1_May_2017 | B | 2 | 9 | | 11 | | 0 | | 20 |
| 4-4_Nov_2016 | B | 2 | 3 | | 29 | | 0 | | 32 |
| 4-4_May_2017 | B | 2 | 3 | | 3 | | 0 | | 6 |
| 4-4_Aug_2020 | B | 2 | 3 | | 9 | | 0 | | 12 |
| 4-5_Aug_2020 | B | 2 | 2 | | 6 | | 0 | | 8 |
| 4-6_May_2017 | B | 2 | 6 | | 6 | | 0 | | 12 |
| 6-4_Jun_2020 | A | 2 | 0 | | 9 | | 7 | | 16 |
| Stump_Apr_2018 | A | 2 | 0 | | 27 | | 7 | | 34 |
| Stump_Mar_2019 | A | 1 | 0 | | 16 | | 0 | | 16 |
| Stump_May_2020 | A | 2 | 0 | | 10 | | 6 | | 16 |
| Laboratory |  | 2 | 2 | | 18 | | 0 | | 20 |
| Total |  |  | 49 | | 272 | | 25 | | 346 |
|  | | | | | | | | | |
| **Locus: Cf4-9A** | Population | No of Alleles | Alleles (bp) | | | | | | |
| Cohort |  |  | 267 | | 282 | | 285 | | Total |
| 1-1_Nov_2016 | A | 1 | 0 | | 20 | | 0 | | 20 |
| 1-1_May_2017 | A | 1 | 0 | | 22 | | 0 | | 22 |
| 1-6_Oct_2019 | A | 1 | 0 | | 20 | | 0 | | 20 |
| 2-2_May_2017 | B | 2 | 0 | | 7 | | 5 | | 12 |
| 3-1_May_2017 | B | 2 | 0 | | 11 | | 5 | | 16 |
| 3-1_Oct_2019 | B | 2 | 0 | | 15 | | 5 | | 20 |
| 3-2_Oct_2019 | B | 2 | 0 | | 13 | | 7 | | 20 |
| 4-1_Nov_2016 | B | 2 | 0 | | 16 | | 8 | | 24 |
| 4-1_May_2017 | B | 2 | 0 | | 14 | | 6 | | 20 |
| 4-4_Nov_2016 | B | 2 | 0 | | 26 | | 6 | | 32 |
| 4-4_May_2017 | B | 2 | 0 | | 5 | | 1 | | 6 |
| 4-4_Aug_2020 | B | 2 | 0 | | 9 | | 3 | | 12 |
| 4-5_Aug_2020 | B | 2 | 0 | | 7 | | 1 | | 8 |
| 4-6_May_2017 | B | 2 | 0 | | 9 | | 3 | | 12 |
| 6-4_Jun_2020 | A | 1 | 0 | | 16 | | 0 | | 16 |
| Stump_Apr_2018 | A | 1 | 0 | | 34 | | 0 | | 34 |
| Stump_Mar_2019 | A | 1 | 0 | | 16 | | 0 | | 16 |
| Stump_May_2020 | A | 1 | 0 | | 16 | | 0 | | 16 |
| Laboratory |  | 2 | 14 | | 6 | | 0 | | 20 |
| Total |  |  | 14 | | 282 | | 50 | | 346 |
|  | | | | | | | | | |
| **Locus: Cf8-4** | Population | No of Alleles | Alleles (bp) | | | | | | |
| Cohort |  |  | 221 | 224 | | 227 | | 284 | Total |
| 1-1_Nov_2016 | A | 2 | 7 | 0 | | 13 | | 0 | 20 |
| 1-1_May_2017 | A | 2 | 6 | 0 | | 16 | | 0 | 22 |
| 1-6_Oct_2019 | A | 2 | 5 | 0 | | 15 | | 0 | 20 |
| 2-2_May_2017 | B | 2 | 0 | 1 | | 11 | | 0 | 12 |
| 3-1_May_2017 | B | 2 | 0 | 4 | | 12 | | 0 | 16 |
| 3-1_Oct_2019 | B | 1 | 0 | 0 | | 20 | | 0 | 20 |
| 3-2_Oct_2019 | B | 1 | 0 | 0 | | 20 | | 0 | 20 |
| 4-1_Nov_2016 | B | 1 | 0 | 0 | | 24 | | 0 | 24 |
| 4-1_May_2017 | B | 1 | 0 | 0 | | 20 | | 0 | 20 |
| 4-4_Nov_2016 | B | 1 | 0 | 0 | | 32 | | 0 | 32 |
| 4-4_May_2017 | B | 1 | 0 | 0 | | 6 | | 0 | 6 |
| 4-4_Aug_2020 | B | 1 | 0 | 0 | | 12 | | 0 | 12 |
| 4-5_Aug_2020 | B | 1 | 0 | 0 | | 8 | | 0 | 8 |
| 4-6_May_2017 | B | 1 | 0 | 0 | | 12 | | 0 | 12 |
| 6-4_Jun_2020 | A | 2 | 5 | 0 | | 11 | | 0 | 16 |
| Stump_Apr_2018 | A | 2 | 9 | 0 | | 25 | | 0 | 34 |
| Stump_Mar_2019 | A | 2 | 7 | 0 | | 9 | | 0 | 16 |
| Stump_May_2020 | A | 2 | 3 | 0 | | 13 | | 0 | 16 |
| Laboratory |  | 2 | 13 | 0 | | 0 | | 7 | 20 |
| Total |  |  | 55 | 5 | | 279 | | 7 | 346 |
|  | | | | | | | | | |
| **Locus: Cf10-5** | Population | No of Alleles | Alleles (bp) | | | | | | |
| Cohort |  |  | 265 | 271 | | 280 | | 283 | Total |
| 1-1_Nov_2016 | A | 2 | 0 | 9 | | 0 | | 11 | 20 |
| 1-1_May_2017 | A | 2 | 0 | 13 | | 0 | | 9 | 22 |
| 1-6_Oct_2019 | A | 2 | 0 | 8 | | 0 | | 12 | 20 |
| 2-2_May_2017 | B | 2 | 4 | 0 | | 0 | | 8 | 12 |
| 3-1_May_2017 | B | 2 | 2 | 0 | | 0 | | 14 | 16 |
| 3-1_Oct_2019 | B | 2 | 8 | 0 | | 0 | | 12 | 20 |
| 3-2_Oct_2019 | B | 2 | 5 | 0 | | 0 | | 15 | 20 |
| 4-1_Nov_2016 | B | 2 | 6 | 0 | | 0 | | 18 | 24 |
| 4-1_May_2017 | B | 2 | 5 | 0 | | 0 | | 15 | 20 |
| 4-4_Nov_2016 | B | 2 | 6 | 0 | | 0 | | 26 | 32 |
| 4-4_May_2017 | B | 2 | 2 | 0 | | 0 | | 4 | 6 |
| 4-4_Aug_2020 | B | 2 | 2 | 0 | | 0 | | 10 | 12 |
| 4-5_Aug_2020 | B | 2 | 2 | 0 | | 0 | | 6 | 8 |
| 4-6_May_2017 | B | 2 | 2 | 0 | | 0 | | 10 | 12 |
| 6-4_Jun_2020 | A | 2 | 0 | 6 | | 0 | | 10 | 16 |
| Stump_Apr_2018 | A | 2 | 0 | 17 | | 0 | | 17 | 34 |
| Stump_Mar_2019 | A | 2 | 0 | 5 | | 0 | | 11 | 16 |
| Stump_May_2020 | A | 2 | 0 | 9 | | 0 | | 7 | 16 |
| Laboratory |  | 2 | 0 | 0 | | 7 | | 13 | 20 |
| Total |  |  | 44 | 67 | | 7 | | 228 | 346 |
