# Supplementary material for: Colony Suppression and Possible Colony Elimination of the Subterranean Termites Coptotermes formosanus and Reticulitermes speratus by Discontinuous Soil Treatment Using a Diluent of Fipronil Suspension Concentrate
Source: Insects. 2021 Apr 8;12(4):334. doi: 10.3390/insects12040334 (PMC8068406; doi:10.3390/insects12040334)
Supplement: Supplementary file 1 [file insects-12-00334-s001.zip › TableS5.docx]

**Table S5:** Allele frequency in each locus of *Reticulitermes speratus* in Isogi Park.

| **Locus: Rs02** | |  | Population | No of Allele | Alleles (bp) | | | | | | | | | | |
| --- | --- | --- | --- | --- | --- | --- | --- | --- | --- | --- | --- | --- | --- | --- | --- |
| Cohort | |  |  |  | 236 | 246 | | | 248 | | 252 | | | 256 | Total |
| 1_1_Oct_2018 | |  | C | 2 | 0 | 8 | | | 0 | | 0 | | | 8 | 16 |
| 1_2_Oct_2018 | |  | C | 2 | 0 | 8 | | | 0 | | 0 | | | 8 | 16 |
| 1_5_Oct_2018 | |  | C | 2 | 0 | 7 | | | 0 | | 0 | | | 7 | 14 |
| 1_5_Mar_2019 | |  | C | 2 | 0 | 8 | | | 0 | | 0 | | | 8 | 16 |
| 1_5_Jun_2019 | |  | C | 2 | 0 | 11 | | | 0 | | 0 | | | 11 | 22 |
| 1_6_Oct_2018 | |  | C | 2 | 0 | 8 | | | 0 | | 0 | | | 8 | 16 |
| 1_7_Oct_2018 | |  | C | 2 | 0 | 8 | | | 0 | | 0 | | | 8 | 16 |
| 1_7_Jun_2019 | |  | C | 2 | 0 | 7 | | | 0 | | 0 | | | 7 | 14 |
| 5_1_May_2020 | |  | D | 3 | 0 | 5 | | | 0 | | 5 | | | 6 | 16 |
| 5_2_May_2020 | |  | D | 3 | 0 | 5 | | | 0 | | 9 | | | 4 | 18 |
| 5_3_May_2020 | |  | D | 3 | 0 | 3 | | | 0 | | 7 | | | 6 | 16 |
| 6_2_May_2020 | |  | D | 3 | 0 | 8 | | | 6 | | 2 | | | 0 | 16 |
| 6_2_Sep_2020 | |  | D | 3 | 0 | 6 | | | 5 | | 5 | | | 0 | 16 |
| 6_3_Sep_2020 | |  | D | 3 | 0 | 11 | | | 3 | | 2 | | | 0 | 16 |
| Laboratory | |  |  | 3 | 8 | 0 | | | 7 | | 0 | | | 9 | 24 |
| Total | |  |  |  | 8 | 103 | | | 21 | | 30 | | | 90 | 252 |
|  |  | | | | | | | | | | | | | | |
| **Locus: Rs03** | |  | Population | No of Allele | Alleles (bp) | | | | | | | | | | |
| Cohort | |  |  |  | 192 | | 194 | | | 196 | | | 198 | | Total |
| 1_1_Oct_2018 | |  | C | 1 | 0 | | 0 | | | 16 | | | 0 | | 16 |
| 1_2_Oct_2018 | |  | C | 1 | 0 | | 0 | | | 16 | | | 0 | | 16 |
| 1_5_Oct_2018 | |  | C | 1 | 0 | | 0 | | | 14 | | | 0 | | 14 |
| 1_5_Mar_2019 | |  | C | 2 | 0 | | 0 | | | 8 | | | 8 | | 16 |
| 1_5_Jun_2019 | |  | C | 2 | 0 | | 0 | | | 15 | | | 7 | | 22 |
| 1_6_Oct_2018 | |  | C | 1 | 0 | | 0 | | | 16 | | | 0 | | 16 |
| 1_7_Oct_2018 | |  | C | 1 | 0 | | 0 | | | 16 | | | 0 | | 16 |
| 1_7_Jun_2019 | |  | C | 2 | 0 | | 0 | | | 10 | | | 4 | | 14 |
| 5_1_May_2020 | |  | D | 2 | 0 | | 0 | | | 15 | | | 1 | | 16 |
| 5_2_May_2020 | |  | D | 1 | 0 | | 0 | | | 18 | | | 0 | | 18 |
| 5_3_May_2020 | |  | D | 1 | 0 | | 0 | | | 16 | | | 0 | | 16 |
| 6_2_May_2020 | |  | D | 1 | 0 | | 0 | | | 16 | | | 0 | | 16 |
| 6_2_Sep_2020 | |  | D | 1 | 0 | | 0 | | | 16 | | | 0 | | 16 |
| 6_3_Sep_2020 | |  | D | 1 | 0 | | 0 | | | 16 | | | 0 | | 16 |
| Laboratory | |  |  | 4 | 4 | | 4 | | | 8 | | | 8 | | 24 |
| Total | |  |  |  | 4 | | 4 | | | 216 | | | 28 | | 252 |
|  |  | | | | | | | | | | | | | | |
| **Locus: Rs05** | |  | Population | No of Allele | Alleles (bp) | | | | | | | | | | |
| Cohort | |  |  |  | 208 | | | 210 | | | | 212 | | | Total |
| 1_1_Oct_2018 | |  | C | 2 | 0 | | | 11 | | | | 5 | | | 16 |
| 1_2_Oct_2018 | |  | C | 2 | 0 | | | 12 | | | | 4 | | | 16 |
| 1_5_Oct_2018 | |  | C | 2 | 0 | | | 12 | | | | 2 | | | 14 |
| 1_5_Mar_2019 | |  | C | 2 | 0 | | | 12 | | | | 4 | | | 16 |
| 1_5_Jun_2019 | |  | C | 2 | 0 | | | 16 | | | | 6 | | | 22 |
| 1_6_Oct_2018 | |  | C | 2 | 0 | | | 13 | | | | 3 | | | 16 |
| 1_7_Oct_2018 | |  | C | 2 | 0 | | | 13 | | | | 3 | | | 16 |
| 1_7_Jun_2019 | |  | C | 2 | 0 | | | 8 | | | | 6 | | | 14 |
| 5_1_May_2020 | |  | D | 1 | 0 | | | 16 | | | | 0 | | | 16 |
| 5_2_May_2020 | |  | D | 1 | 0 | | | 18 | | | | 0 | | | 18 |
| 5_3_May_2020 | |  | D | 1 | 0 | | | 16 | | | | 0 | | | 16 |
| 6_2_May_2020 | |  | D | 1 | 0 | | | 16 | | | | 0 | | | 16 |
| 6_2_Sep_2020 | |  | D | 1 | 0 | | | 16 | | | | 0 | | | 16 |
| 6_3_Sep_2020 | |  | D | 1 | 0 | | | 16 | | | | 0 | | | 16 |
| Laboratory | |  |  | 3 | 11 | | | 7 | | | | 6 | | | 24 |
| Total | |  |  |  | 11 | | | 202 | | | | 39 | | | 252 |
|  |  | | | | | | | | | | | | | | |
| **Locus: Rs07** | |  | Population | No of Allele | Alleles (bp) | | | | | | | | | | |
| Cohort | |  |  |  | 190 | | | | | 192 | | | | | Total |
| 1_1_Oct_2018 | |  | C | 1 | 0 | | | | | 16 | | | | | 16 |
| 1_2_Oct_2018 | |  | C | 2 | 2 | | | | | 14 | | | | | 16 |
| 1_5_Oct_2018 | |  | C | 2 | 6 | | | | | 8 | | | | | 14 |
| 1_5_Mar_2019 | |  | C | 1 | 0 | | | | | 16 | | | | | 16 |
| 1_5_Jun_2019 | |  | C | 1 | 0 | | | | | 22 | | | | | 22 |
| 1_6_Oct_2018 | |  | C | 2 | 8 | | | | | 8 | | | | | 16 |
| 1_7_Oct_2018 | |  | C | 2 | 2 | | | | | 14 | | | | | 16 |
| 1_7_Jun_2019 | |  | C | 2 | 2 | | | | | 12 | | | | | 14 |
| 5_1_May_2020 | |  | D | 1 | 0 | | | | | 16 | | | | | 16 |
| 5_2_May_2020 | |  | D | 1 | 0 | | | | | 18 | | | | | 18 |
| 5_3_May_2020 | |  | D | 1 | 0 | | | | | 16 | | | | | 16 |
| 6_2_May_2020 | |  | D | 1 | 0 | | | | | 16 | | | | | 16 |
| 6_2_Sep_2020 | |  | D | 1 | 0 | | | | | 16 | | | | | 16 |
| 6_3_Sep_2020 | |  | D | 1 | 0 | | | | | 16 | | | | | 16 |
| Laboratory | |  |  | 2 | 2 | | | | | 22 | | | | | 24 |
| Total | |  |  |  | 22 | | | | | 230 | | | | | 252 |
