# Supplementary material for: Colony Suppression and Possible Colony Elimination of the Subterranean Termites Coptotermes formosanus and Reticulitermes speratus by Discontinuous Soil Treatment Using a Diluent of Fipronil Suspension Concentrate
Source: Insects. 2021 Apr 8;12(4):334. doi: 10.3390/insects12040334 (PMC8068406; doi:10.3390/insects12040334)
Supplement: Supplementary file 1 [file insects-12-00334-s001.zip › TableS6.docx]

**Table S6:** Allele frequency in each locus of *Reticulitermes speratus* at Kindai University.

| **Locus: Rs02** | Population | No of Allele | Alleles (bp) | | | | | | | | | | | | | | | |
| --- | --- | --- | --- | --- | --- | --- | --- | --- | --- | --- | --- | --- | --- | --- | --- | --- | --- | --- |
| Cohort |  |  | 236 | 244 | | 246 | 247 | | 248 | | 250 | | 252 | | 254 | | 256 | Total |
| 1_Oct_2015 | D | 2 | 0 | 0 | | 10 | 0 | | 8 | | 0 | | 0 | | 0 | | 0 | 18 |
| 1_Nov_2018 | B | 1 | 0 | 0 | | 0 | 0 | | 16 | | 0 | | 0 | | 0 | | 0 | 16 |
| 1_Dec_2018 | B | 1 | 0 | 0 | | 0 | 0 | | 16 | | 0 | | 0 | | 0 | | 0 | 16 |
| 1_May_2019 | B | 1 | 0 | 0 | | 0 | 0 | | 24 | | 0 | | 0 | | 0 | | 0 | 24 |
| 1_Nov_2019 | B | 3 | 0 | 0 | | 0 | 0 | | 15 | | 0 | | 3 | | 0 | | 4 | 22 |
| 2_Dec_2014 | C | 3 | 0 | 0 | | 9 | 0 | | 14 | | 0 | | 1 | | 0 | | 0 | 24 |
| 2_Nov_2019 | B | 2 | 0 | 0 | | 1 | 0 | | 23 | | 0 | | 0 | | 0 | | 0 | 24 |
| 3_Nov_2018 | B | 1 | 0 | 0 | | 0 | 0 | | 16 | | 0 | | 0 | | 0 | | 0 | 16 |
| 3_Dec_2018 | B | 1 | 0 | 0 | | 0 | 0 | | 16 | | 0 | | 0 | | 0 | | 0 | 16 |
| 3_May_2019 | B | 2 | 0 | 0 | | 2 | 0 | | 22 | | 0 | | 0 | | 0 | | 0 | 24 |
| 4_Oct_2015 | A | 3 | 0 | 0 | | 7 | 0 | | 9 | | 0 | | 0 | | 0 | | 12 | 28 |
| 5_Dec_2014 | D | 2 | 0 | 0 | | 10 | 0 | | 14 | | 0 | | 0 | | 0 | | 0 | 24 |
| 5_Oct_2015 | D | 2 | 0 | 0 | | 19 | 0 | | 15 | | 0 | | 0 | | 0 | | 0 | 34 |
| 5_Nov_2018 | B | 1 | 0 | 0 | | 0 | 0 | | 16 | | 0 | | 0 | | 0 | | 0 | 16 |
| 6_Oct_2015 | D | 2 | 0 | 0 | | 30 | 0 | | 8 | | 0 | | 0 | | 0 | | 0 | 38 |
| 6_Nov_2019 | B | 2 | 0 | 0 | | 6 | 0 | | 14 | | 0 | | 0 | | 0 | | 0 | 20 |
| 7_Oct_2015 | A | 3 | 0 | 0 | | 1 | 0 | | 5 | | 2 | | 0 | | 0 | | 0 | 8 |
| 7_Nov_2017 | B | 2 | 0 | 0 | | 4 | 0 | | 20 | | 0 | | 0 | | 0 | | 0 | 24 |
| 8_Nov_2019 | E | 2 | 0 | 0 | | 0 | 0 | | 2 | | 0 | | 0 | | 0 | | 14 | 16 |
| 9_May_2016 | B | 2 | 0 | 0 | | 1 | 0 | | 23 | | 0 | | 0 | | 0 | | 0 | 24 |
| 11_May_2019 | A | 3 | 0 | 0 | | 5 | 0 | | 16 | | 0 | | 0 | | 0 | | 1 | 22 |
| 12_May_2016 | C | 2 | 0 | 9 | | 0 | 0 | | 15 | | 0 | | 0 | | 0 | | 0 | 24 |
| 13_Dec_2014 | C | 2 | 0 | 0 | | 0 | 0 | | 11 | | 0 | | 1 | | 0 | | 0 | 12 |
| 13_Oct_2015 | C | 4 | 0 | 0 | | 0 | 1 | | 33 | | 0 | | 1 | | 0 | | 1 | 36 |
| 14_May_2019 | A | 3 | 0 | 0 | | 4 | 0 | | 14 | | 0 | | 0 | | 0 | | 6 | 24 |
| 15_Dec_2018 | A | 3 | 0 | 0 | | 1 | 0 | | 9 | | 0 | | 0 | | 0 | | 6 | 16 |
| 16_Sept_2013 | A | 2 | 0 | 0 | | 0 | 0 | | 13 | | 0 | | 0 | | 0 | | 15 | 28 |
| 16_Dec_2018 | A | 3 | 0 | 0 | | 2 | 0 | | 11 | | 0 | | 0 | | 0 | | 3 | 16 |
| 16_May_2019 | A | 3 | 0 | 0 | | 3 | 0 | | 13 | | 0 | | 0 | | 0 | | 6 | 22 |
| 18_Aug_2018 | A | 3 | 0 | 0 | | 3 | 0 | | 11 | | 0 | | 0 | | 0 | | 8 | 22 |
| 18_Dec_2018 | - | 3 | 0 | 0 | | 6 | 0 | | 0 | | 0 | | 0 | | 4 | | 6 | 16 |
| 18_Nov_2019 | E | 3 | 0 | 0 | | 5 | 0 | | 0 | | 0 | | 0 | | 2 | | 15 | 22 |
| 20_Aug_2018 | E | 3 | 0 | 0 | | 3 | 0 | | 0 | | 0 | | 0 | | 6 | | 11 | 20 |
| 27_Aug_2018 | A | 3 | 0 | 0 | | 3 | 0 | | 8 | | 0 | | 0 | | 0 | | 11 | 22 |
| Laboratory |  | 3 | 8 | 0 | | 0 | 0 | | 7 | | 0 | | 0 | | 0 | | 9 | 24 |
| Total |  |  | 8 | 9 | | 135 | 1 | | 457 | | 2 | | 6 | | 12 | | 128 | 758 |
|  | | | | | | | | | | | | | | | | | | |
| **Locus: Rs03** | Population | No of Allele | Alleles (bp) | | | | | | | | | | | | | | | |
| Cohort |  |  | 192 | | 194 | | 196 | | | 198 | | | 200 | | | 202 | | Total |
| 1_Oct_2015 | D | 3 | 4 | | 0 | | 6 | | | 0 | | | 8 | | | 0 | | 18 |
| 1_Nov_2018 | B | 2 | 0 | | 0 | | 4 | | | 12 | | | 0 | | | 0 | | 16 |
| 1_Dec_2018 | B | 2 | 0 | | 0 | | 7 | | | 9 | | | 0 | | | 0 | | 16 |
| 1_May_2019 | B | 2 | 0 | | 0 | | 2 | | | 22 | | | 0 | | | 0 | | 24 |
| 1_Nov_2019 | B | 2 | 0 | | 0 | | 4 | | | 18 | | | 0 | | | 0 | | 22 |
| 2_Dec_2014 | C | 3 | 0 | | 8 | | 0 | | | 9 | | | 0 | | | 7 | | 24 |
| 2_Nov_2019 | B | 2 | 0 | | 0 | | 6 | | | 18 | | | 0 | | | 0 | | 24 |
| 3_Nov_2018 | B | 2 | 0 | | 0 | | 6 | | | 10 | | | 0 | | | 0 | | 16 |
| 3_Dec_2018 | B | 2 | 0 | | 0 | | 7 | | | 9 | | | 0 | | | 0 | | 16 |
| 3_May_2019 | B | 2 | 0 | | 0 | | 5 | | | 19 | | | 0 | | | 0 | | 24 |
| 4_Oct_2015 | A | 3 | 0 | | 0 | | 2 | | | 25 | | | 1 | | | 0 | | 28 |
| 5_Dec_2014 | D | 3 | 4 | | 0 | | 16 | | | 0 | | | 4 | | | 0 | | 24 |
| 5_Oct_2015 | D | 3 | 7 | | 0 | | 15 | | | 0 | | | 12 | | | 0 | | 34 |
| 5_Nov_2018 | B | 2 | 0 | | 0 | | 6 | | | 10 | | | 0 | | | 0 | | 16 |
| 6_Oct_2015 | D | 3 | 2 | | 0 | | 25 | | | 0 | | | 11 | | | 0 | | 38 |
| 6_Nov_2019 | B | 2 | 0 | | 0 | | 2 | | | 18 | | | 0 | | | 0 | | 20 |
| 7_Oct_2015 | A | 3 | 0 | | 0 | | 2 | | | 5 | | | 1 | | | 0 | | 8 |
| 7_Nov_2017 | B | 2 | 0 | | 0 | | 1 | | | 23 | | | 0 | | | 0 | | 24 |
| 8_Nov_2019 | E | 2 | 0 | | 0 | | 0 | | | 11 | | | 0 | | | 5 | | 16 |
| 9_May_2016 | B | 2 | 0 | | 0 | | 4 | | | 20 | | | 0 | | | 0 | | 24 |
| 11_May_2019 | A | 2 | 0 | | 0 | | 5 | | | 17 | | | 0 | | | 0 | | 22 |
| 12_May_2016 | C | 2 | 0 | | 0 | | 13 | | | 11 | | | 0 | | | 0 | | 24 |
| 13_Dec_2014 | C | 3 | 0 | | 2 | | 0 | | | 6 | | | 0 | | | 4 | | 12 |
| 13_Oct_2015 | C | 2 | 0 | | 0 | | 0 | | | 24 | | | 0 | | | 12 | | 36 |
| 14_May_2019 | A | 2 | 0 | | 0 | | 9 | | | 15 | | | 0 | | | 0 | | 24 |
| 15_Dec_2018 | A | 2 | 0 | | 0 | | 9 | | | 7 | | | 0 | | | 0 | | 16 |
| 16_Sept_2013 | A | 3 | 0 | | 0 | | 11 | | | 7 | | | 0 | | | 10 | | 28 |
| 16_Dec_2018 | A | 2 | 0 | | 0 | | 9 | | | 7 | | | 0 | | | 0 | | 16 |
| 16_May_2019 | A | 2 | 0 | | 0 | | 6 | | | 16 | | | 0 | | | 0 | | 22 |
| 18_Aug_2018 | A | 2 | 0 | | 0 | | 5 | | | 17 | | | 0 | | | 0 | | 22 |
| 18_Dec_2018 | - | 2 | 0 | | 0 | | 8 | | | 8 | | | 0 | | | 0 | | 16 |
| 18_Nov_2019 | E | 1 | 0 | | 0 | | 0 | | | 22 | | | 0 | | | 0 | | 22 |
| 20_Aug_2018 | E | 1 | 0 | | 0 | | 0 | | | 20 | | | 0 | | | 0 | | 20 |
| 27_Aug_2018 | A | 2 | 0 | | 0 | | 11 | | | 11 | | | 0 | | | 0 | | 22 |
| Laboratory |  | 4 | 4 | | 4 | | 8 | | | 8 | | | 0 | | | 0 | | 24 |
| Total |  |  | 21 | | 14 | | 214 | | | 434 | | | 37 | | | 38 | | 758 |
|  | | | | | | | | | | | | | | | | | | |
| **Locus: Rs05** | Population | No of Allele | Alleles (bp) | | | | | | | | | | | | | | | |
| Cohort |  |  | 208 | | | 210 | | 212 | | | | 214 | | | | 218 | | Total |
| 1_Oct_2015 | D | 1 | 0 | | | 18 | | 0 | | | | 0 | | | | 0 | | 18 |
| 1_Nov_2018 | B | 1 | 0 | | | 16 | | 0 | | | | 0 | | | | 0 | | 16 |
| 1_Dec_2018 | B | 1 | 0 | | | 16 | | 0 | | | | 0 | | | | 0 | | 16 |
| 1_May_2019 | B | 1 | 0 | | | 24 | | 0 | | | | 0 | | | | 0 | | 24 |
| 1_Nov_2019 | B | 1 | 0 | | | 22 | | 0 | | | | 0 | | | | 0 | | 22 |
| 2_Dec_2014 | C | 1 | 0 | | | 24 | | 0 | | | | 0 | | | | 0 | | 24 |
| 2_Nov_2019 | B | 1 | 0 | | | 24 | | 0 | | | | 0 | | | | 0 | | 24 |
| 3_Nov_2018 | B | 1 | 0 | | | 16 | | 0 | | | | 0 | | | | 0 | | 16 |
| 3_Dec_2018 | B | 1 | 0 | | | 16 | | 0 | | | | 0 | | | | 0 | | 16 |
| 3_May_2019 | B | 1 | 0 | | | 24 | | 0 | | | | 0 | | | | 0 | | 24 |
| 4_Oct_2015 | A | 2 | 0 | | | 22 | | 0 | | | | 6 | | | | 0 | | 28 |
| 5_Dec_2014 | D | 1 | 0 | | | 24 | | 0 | | | | 0 | | | | 0 | | 24 |
| 5_Oct_2015 | D | 1 | 0 | | | 34 | | 0 | | | | 0 | | | | 0 | | 34 |
| 5_Nov_2018 | B | 1 | 0 | | | 16 | | 0 | | | | 0 | | | | 0 | | 16 |
| 6_Oct_2015 | D | 1 | 0 | | | 38 | | 0 | | | | 0 | | | | 0 | | 38 |
| 6_Nov_2019 | B | 1 | 0 | | | 20 | | 0 | | | | 0 | | | | 0 | | 20 |
| 7_Oct_2015 | A | 3 | 0 | | | 4 | | 0 | | | | 3 | | | | 1 | | 8 |
| 7_Nov_2017 | B | 1 | 0 | | | 24 | | 0 | | | | 0 | | | | 0 | | 24 |
| 8_Nov_2019 | E | 2 | 0 | | | 12 | | 0 | | | | 4 | | | | 0 | | 16 |
| 9_May_2016 | B | 1 | 0 | | | 24 | | 0 | | | | 0 | | | | 0 | | 24 |
| 11_May_2019 | A | 2 | 0 | | | 15 | | 0 | | | | 7 | | | | 0 | | 22 |
| 12_May_2016 | C | 2 | 0 | | | 24 | | 0 | | | | 0 | | | | 0 | | 24 |
| 13_Dec_2014 | C | 1 | 0 | | | 12 | | 0 | | | | 0 | | | | 0 | | 12 |
| 13_Oct_2015 | C | 1 | 0 | | | 36 | | 0 | | | | 0 | | | | 0 | | 36 |
| 14_May_2019 | A | 2 | 0 | | | 18 | | 0 | | | | 6 | | | | 0 | | 24 |
| 15_Dec_2018 | A | 2 | 0 | | | 11 | | 0 | | | | 5 | | | | 0 | | 16 |
| 16_Sept_2013 | A | 1 | 0 | | | 28 | | 0 | | | | 0 | | | | 0 | | 28 |
| 16_Dec_2018 | A | 2 | 0 | | | 15 | | 0 | | | | 1 | | | | 0 | | 16 |
| 16_May_2019 | A | 2 | 0 | | | 18 | | 0 | | | | 4 | | | | 0 | | 22 |
| 18_Aug_2018 | A | 2 | 0 | | | 17 | | 0 | | | | 5 | | | | 0 | | 22 |
| 18_Dec_2018 | - | 1 | 0 | | | 16 | | 0 | | | | 0 | | | | 0 | | 16 |
| 18_Nov_2019 | E | 1 | 0 | | | 22 | | 0 | | | | 0 | | | | 0 | | 22 |
| 20_Aug_2018 | E | 1 | 0 | | | 20 | | 0 | | | | 0 | | | | 0 | | 20 |
| 27_Aug_2018 | A | 2 | 0 | | | 20 | | 0 | | | | 2 | | | | 0 | | 22 |
| Laboratory |  | 3 | 11 | | | 7 | | 6 | | | | 0 | | | | 0 | | 24 |
| Total |  |  | 11 | | | 697 | | 6 | | | | 43 | | | | 1 | | 758 |
|  | | | | | | | | | | | | | | | | | | |
| **Locus: Rs07** | Population | No of Allele | Alleles (bp) | | | | | | | | | | | | | | | |
| Cohort |  |  | 188 | | | 190 | | | | 192 | | | | 194 | | | | Total |
| 1_Oct_2015 | D | 1 | 0 | | | 0 | | | | 18 | | | | 0 | | | | 18 |
| 1_Nov_2018 | B | 1 | 0 | | | 0 | | | | 16 | | | | 0 | | | | 16 |
| 1_Dec_2018 | B | 1 | 0 | | | 0 | | | | 16 | | | | 0 | | | | 16 |
| 1_May_2019 | B | 1 | 0 | | | 0 | | | | 24 | | | | 0 | | | | 24 |
| 1_Nov_2019 | B | 1 | 0 | | | 0 | | | | 22 | | | | 0 | | | | 22 |
| 2_Dec_2014 | C | 2 | 0 | | | 12 | | | | 12 | | | | 0 | | | | 24 |
| 2_Nov_2019 | B | 1 | 0 | | | 0 | | | | 24 | | | | 0 | | | | 24 |
| 3_Nov_2018 | B | 1 | 0 | | | 0 | | | | 16 | | | | 0 | | | | 16 |
| 3_Dec_2018 | B | 1 | 0 | | | 0 | | | | 16 | | | | 0 | | | | 16 |
| 3_May_2019 | B | 1 | 0 | | | 0 | | | | 24 | | | | 0 | | | | 24 |
| 4_Oct_2015 | A | 1 | 0 | | | 0 | | | | 28 | | | | 0 | | | | 28 |
| 5_Dec_2014 | D | 1 | 0 | | | 0 | | | | 24 | | | | 0 | | | | 24 |
| 5_Oct_2015 | D | 1 | 0 | | | 0 | | | | 34 | | | | 0 | | | | 34 |
| 5_Nov_2018 | B | 1 | 0 | | | 0 | | | | 16 | | | | 0 | | | | 16 |
| 6_Oct_2015 | D | 1 | 0 | | | 0 | | | | 38 | | | | 0 | | | | 38 |
| 6_Nov_2019 | B | 1 | 0 | | | 0 | | | | 20 | | | | 0 | | | | 20 |
| 7_Oct_2015 | A | 2 | 0 | | | 0 | | | | 6 | | | | 2 | | | | 8 |
| 7_Nov_2017 | B | 1 | 0 | | | 0 | | | | 24 | | | | 0 | | | | 24 |
| 8_Nov_2019 | E | 1 | 0 | | | 0 | | | | 16 | | | | 0 | | | | 16 |
| 9_May_2016 | B | 1 | 0 | | | 0 | | | | 24 | | | | 0 | | | | 24 |
| 11_May_2019 | A | 1 | 0 | | | 0 | | | | 22 | | | | 0 | | | | 22 |
| 12_May_2016 | C | 2 | 0 | | | 15 | | | | 9 | | | | 0 | | | | 24 |
| 13_Dec_2014 | C | 2 | 0 | | | 6 | | | | 6 | | | | 0 | | | | 12 |
| 13_Oct_2015 | C | 2 | 0 | | | 9 | | | | 27 | | | | 0 | | | | 36 |
| 14_May_2019 | A | 1 | 0 | | | 0 | | | | 24 | | | | 0 | | | | 24 |
| 15_Dec_2018 | A | 1 | 0 | | | 0 | | | | 16 | | | | 0 | | | | 16 |
| 16_Sept_2013 | A | 1 | 0 | | | 0 | | | | 28 | | | | 0 | | | | 28 |
| 16_Dec_2018 | A | 1 | 0 | | | 0 | | | | 16 | | | | 0 | | | | 16 |
| 16_May_2019 | A | 1 | 0 | | | 0 | | | | 22 | | | | 0 | | | | 22 |
| 18_Aug_2018 | A | 1 | 0 | | | 0 | | | | 22 | | | | 0 | | | | 22 |
| 18_Dec_2018 | - | 2 | 6 | | | 0 | | | | 10 | | | | 0 | | | | 16 |
| 18_Nov_2019 | E | 1 | 0 | | | 0 | | | | 22 | | | | 0 | | | | 22 |
| 20_Aug_2018 | E | 2 | 5 | | | 0 | | | | 15 | | | | 0 | | | | 20 |
| 27_Aug_2018 | A | 1 | 0 | | | 0 | | | | 22 | | | | 0 | | | | 22 |
| Laboratory |  | 2 | 0 | | | 2 | | | | 22 | | | | 0 | | | | 24 |
| Total |  |  | 11 | | | 44 | | | | 701 | | | | 2 | | | | 758 |
